# Childhood asthma and the microbiome: from gut-lung axis mechanisms to precision prevention strategies

**DOI:** 10.3389/fimmu.2026.1902053

**Published:** 2026-09-02

**Authors:** Xuan Liang, De Chen, Jiafen Hu, Li Guo, Hui Yang, Qi Guo, Rongfang Zhang

**Affiliations:** 1 Department of Allergy, Gansu Provincial Maternity and Child Care Hospital, Lanzhou, Gansu, China; 2 Emergency Medical Center, Gansu Provincial Maternity and Child Care Hospital, Lanzhou, Gansu, China

**Keywords:** childhood asthma, microbiome, gut–lung axis, respiratory microbiome, gut microbiome, precision prevention

## Abstract

Childhood asthma is a highly heterogeneous chronic respiratory disease, and its onset and progression are intricately linked to genetic susceptibility, environmental exposure, immune development, and the establishment of the early-life microbiome. In recent years, studies on the gut and respiratory microbiomes have suggested that the composition, metabolic functions, and interactions of microbial communities with the host immune system may be involved in the formation of asthma susceptibility, shaping of inflammatory phenotypes, and disease progression in children. The gut-lung axis, as an important pathway connecting gut microbiome, respiratory immunity, and systemic inflammatory responses, provides a new perspective for understanding the early mechanisms of childhood asthma. This article reviews the characteristics of the respiratory and gut microbiomes associated with childhood asthma, with a focus on the roles of the gut-lung axis, microbial metabolites, mucosal immune regulation, and environmental exposure. It also evaluates the research progress of probiotics, prebiotics, nutritional interventions, and novel microecological therapies. Additionally, the potential of microbial maturity, microbial metabolites, and immunophenotypes as biomarkers for risk prediction, phenotype stratification, and treatment response is analyzed. Furthermore, the role of multi-omics integration in supporting the identification of responsive populations, matching of intervention strategies, and dynamic monitoring of efficacy is discussed. Current evidence suggests that the microbiome offers promising targets for risk assessment and precision prevention of childhood asthma. However, relevant research still faces challenges such as ambiguous causality, high cohort heterogeneity, limited reproducibility of candidate biomarkers, inconsistent intervention outcomes, and insufficient evidence of long-term safety. At present, most biomarkers and multi-omics models remain in the stage of association discovery, lacking unified thresholds, cross-cohort validation, and biomarker-guided randomized controlled trials in children. Therefore, they cannot be routinely used for patient stratification or intervention selection. Future efforts should rely on standardized longitudinal birth cohorts, multi-omics integration, external validation, and high-quality clinical trials to clarify the incremental value of microbiome biomarkers over traditional clinical indicators and their clinical utility in the individualized management of childhood asthma.

## 1 Introduction

Childhood asthma is a common and highly heterogeneous chronic respiratory disease, primarily characterized by recurrent wheezing, coughing, shortness of breath, and airway hyperresponsiveness, which can have long-term effects on children’s growth and development, quality of life, and family caregiving burdens. Its occurrence and development result from the combined effects of genetic susceptibility, environmental exposure, respiratory infections, immune development, and host metabolic status (1, 2). There are significant differences among children in terms of age of onset, allergic status, inflammatory phenotype, risk of acute exacerbations, and treatment response (3, 4). Traditional studies have mainly explained the occurrence of childhood asthma from aspects such as genetic background, allergic constitution, viral infections, air pollution, antibiotic exposure, delivery methods, and feeding patterns (5). However, these factors still cannot fully explain the differences in disease susceptibility, clinical phenotype, and long-term prognosis among affected children. Particularly in early life, when the immune system, mucosal barrier, and microbiome are all in a rapid developmental stage, environmental exposure may alter children’s responses to allergens, viral infections, and pollutants by influencing immune shaping and barrier maturation (6, 7).

In recent years, microbiome research has provided new perspectives for understanding the heterogeneity of childhood asthma. The gut and respiratory microbiota are gradually established in early life and participate in immune tolerance, mucosal barrier maintenance, inflammatory regulation, and metabolite production (8). Existing studies suggest that imbalances in the gut or respiratory microbial communities may be involved in the formation of childhood asthma susceptibility, shaping of inflammatory phenotypes, and disease progression. Gut microbes and their products, such as short-chain fatty acids (SCFAs), tryptophan metabolites, and bile acids, may influence the lung microenvironment through systemic circulation and immune signaling pathways (9, 10). Concurrently, changes in the respiratory microbiota may be associated with susceptibility to viral infections, airway inflammation, acute exacerbations, and treatment response (11). The resulting concept of the gut-lung axis provides a candidate mechanistic framework for integrating the gut microbiome, respiratory microbiome, and host immune response. However, existing studies are inconsistent regarding microbial characteristics, causal direction, and clinical significance. The observed microbial changes may either be involved in disease pathogenesis or be the result of infection, inflammation, treatment, or shared environmental exposure.

This article summarizes the characteristics of the respiratory and gut microbiome associated with childhood asthma, the developmental trajectory of the microbiome in early life, the immune-metabolic mechanisms mediated by the gut-lung axis, and the impact of environmental exposure and microbiome interventions. Unlike previous reviews that primarily focused on changes in microbial composition or a single gut-lung axis mechanism, the main added value of this article lies in integrating early-life microbiome development, respiratory and gut microbiota, microbial function, host immune-metabolic responses, clinical phenotypes, and intervention evidence within a unified evaluation framework. It also assesses the causal credibility and clinical translation boundaries based on different study designs and levels of evidence maturity. On this basis, this article compares the evidence levels of observational studies, animal experiments, and clinical intervention studies, analyzes potential sources of inconsistency in research findings, and clearly distinguishes between relatively robust clinical associations, mechanistically plausible but yet-to-be-validated hypotheses, and intervention strategies that are still in the exploratory stage and insufficient to support routine clinical application. Furthermore, this article evaluates the potential of the microbiome, metabolites, and immune phenotypes in risk stratification, identification of beneficiary populations, prognosis assessment, and efficacy monitoring. In doing so, it aims to establish an evidence chain linking “microbiome development-functional changes-immune-metabolic mechanisms-clinical phenotypes-precision intervention,” thereby providing a more critical and translation-oriented evidence framework for the precision prevention and individualized management of childhood asthma.

## 2 Microbiome characteristics associated with childhood asthma

### 2.1 Upper respiratory microbiome

The upper respiratory microbiome in children is gradually established early in life, and its composition and dynamic changes may be associated with the risk of subsequent wheezing, asthma development, and acute exacerbations (12). Studies have shown that the microbial communities in the nasal cavity and nasopharynx of infants are often dominated by genera such as *Moraxella, Haemophilus, Streptococcus, Corynebacterium*, and *Dolosigranulum*, with their community structure influenced by multiple factors including age, mode of delivery, feeding method, antibiotic exposure, viral infections, and environmental factors (13, 14). Some potentially inflammation-associated bacteria, such as *Moraxella, Haemophilus*, and *Streptococcus*, can exist as normal colonizers in the upper respiratory tract of children but may also be associated with recurrent wheezing, acute exacerbations, and airway inflammation in the context of specific ages, viral infections, or host immunity (15–19). Among these, a nasopharyngeal community dominated by *Moraxella* in infancy may be associated with an increased risk of subsequent asthma, but current evidence is insufficient to determine whether these changes are a driving factor of inflammation, opportunistic expansion following viral infection, or a concomitant marker of altered airway ecology (20).

Conversely, genera such as *Corynebacterium* and *Dolosigranulum* have been associated with a lower risk of wheezing or asthma in some studies, suggesting a potential protective role (21, 22). This association may involve maintaining local ecological stability, competitively inhibiting potentially inflammation-associated bacteria, or modulating host immunity, but direct mechanistic evidence remains limited. Furthermore, different strains within the same genus can exhibit significant differences in virulence, adhesion capacity, and metabolic function, making it inappropriate to define an entire genus as “harmful” or “beneficial.”

The relationship between upper respiratory microbial diversity and childhood asthma is inconsistent. Some studies have found reduced diversity in children with asthma or wheezing, while others have observed increased diversity or altered community structure (23). Reduced diversity may reflect a shift from a relatively balanced community state to one dominated by a few taxa, accompanied by decreased community stability and colonization resistance (24). Under viral infection or other inflammatory stimuli, this ecological vulnerability may favor the expansion of potentially inflammation-associated bacteria, enhancing epithelial damage, innate immune activation, and granulocytic inflammation, thereby contributing to recurrent wheezing or susceptibility to acute exacerbations (25). However, higher diversity does not necessarily indicate a healthy state, as it may result from contamination by oropharyngeal microbes, transient expansion during infection, or coexistence of abnormal flora. Therefore, the clinical significance of diversity depends on age, community composition, virus-bacteria co-occurrence status, and sampling time point, and cannot be interpreted in isolation from the specific ecological context (26). Compared with single genera or overall diversity, bacterial combinations, community maturation trajectories, and functional characteristics may offer greater explanatory power, while single upper respiratory genera are currently insufficient as stable biomarkers for asthma diagnosis or risk stratification (27).

### 2.2 Lower respiratory microbiome

Historically, the lower respiratory tract was considered to be nearly sterile. However, with the development of high-throughput sequencing technology, an increasing number of studies have shown that the lower respiratory tract may harbor low-biomass microbial communities with potential biological significance (28). Compared to the upper respiratory tract, research on the lower respiratory tract microbiome in children is more challenging. Due to ethical and operational constraints in obtaining lower respiratory tract samples from children, existing studies often rely on induced sputum, bronchoalveolar lavage fluid (BAL), or other indirect samples (29). These samples represent different anatomical sites and biological information: BAL is relatively close to the distal airways but is typically obtained only from children with clinical indications; induced sputum contains varying degrees of oropharyngeal and proximal airway components. Therefore, BAL, induced sputum, and oropharyngeal samples cannot be considered interchangeable “lower respiratory tract samples.” When comparing overall community structure, some studies further cluster samples based on microbial composition and relative abundance patterns, classifying those with similar ecological characteristics into different “microbial community subtypes.” These subtypes are usually named after dominant genera or characteristic flora, such as *Haemophilus-dominant* or *Neisseria*-dominant communities, which are ecological classifications constructed in research rather than standardized clinical classifications for childhood asthma.

Some lower respiratory tract studies have observed community alterations dominated by Proteobacteria-related flora, including representative genera such as *Haemophilus, Neisseria, Moraxella*, and *Streptococcus*, and have found that certain microbial community subtypes dominated by these genera are associated with decreased lung function, granulocytic inflammatory phenotypes, or disease severity (30–32). In contrast to analyses of single genera, community subtypes can simultaneously reflect dominant bacteria, co-occurring flora, and overall ecological structure, thus potentially providing a more comprehensive description of the airway microecological state than single taxonomic units. However, their stability and cross-cohort reproducibility are still influenced by factors such as sampling site, study population, clustering methods, and analytical workflows.

These genera exhibit significant overlap with common colonizing bacteria in the upper respiratory tract; therefore, determining whether they truly originate from the distal airways requires judgment based on sample type and contamination control. Lower respiratory tract samples have low biomass, and trace reagent contamination, environmental background DNA, and oropharyngeal carriage can all generate sequencing signals comparable to or even stronger than those from true lung signals (33). Therefore, studies should simultaneously report sampling routes, sample biomass, sequencing depth, blank extraction controls, reagent negative controls, and correction for contaminating sequences, and, where possible, interpret results in conjunction with absolute bacterial load. Without these controls, defining lower respiratory tract flora or microbial community subtypes solely based on relative abundance differences may erroneously attribute technical noise or upper respiratory tract contamination to distal airway ecological features. Consequently, the community subtypes identified in different studies are currently more suitable as research tools for describing airway ecological heterogeneity, and their clinical stability, cross-cohort consistency, and risk stratification value still require further validation.

Invasive pediatric samples also exhibit significant selection bias. Children undergoing bronchoscopy or BAL are typically those with more severe disease, complex diagnoses, or poor treatment responses, which may lead to overrepresentation of severe asthma and specific inflammatory phenotypes in studies, limiting the generalizability of results to children with typical asthma. Additionally, inhaled corticosteroids, antibiotics, and acute exacerbation treatments can alter local flora structure (34). Since disease severity is closely related to treatment intensity, the association between flora characteristics and clinical outcomes may be partially confounded by treatment effects. Therefore, the key to lower respiratory tract research is not to continue adding lists of differential genera, but to confirm the anatomical source of observed signals, their ecological stability, and their independent significance relative to sampling methods and treatment exposure.

### 2.3 Gut microbiome

The gut microbiome is an important component of early immune and metabolic development in children, and its maturation process may be closely related to asthma susceptibility. The establishment of the gut microbiome early in life is influenced by factors such as mode of delivery, breastfeeding, introduction of solid foods, antibiotic exposure, home environment, and dietary patterns (35, 36). Studies suggest that insufficient gut microbial diversity early after birth, delayed community maturation, or imbalances in specific bacterial proportions may be associated with an increased risk of subsequent wheezing, allergic diseases, and asthma (37, 38).

Regarding specific bacteria, some studies have reported that in children at high risk for asthma or those who subsequently develop wheezing, the relative abundance of *Faecalibacterium, Lachnospira*, and some members of *Ruminococcus*, which have potential SCFAs-producing capacity, is reduced. Additionally, a lower abundance of *Bifidobacterium* has also been associated with increased risk of allergy or wheezing in some early-life cohorts (39–41). In contrast, the direction of association for *Bacteroides* is inconsistent across studies, possibly influenced by species or strain differences, mode of delivery, diet, and geographical background. Therefore, the same genus may show increased, decreased, or no significant differences in different studies, and its function or pathogenic significance cannot be inferred solely from the genus name (42).

A reduction in potentially SCFAs-producing bacteria may suggest insufficient fermentation function and immune tolerance-related metabolic capacity, while relative enrichment of certain bacteria may reflect changes in dietary substrates, an inflammatory environment, or a decrease in other commensals. However, an increase in relative abundance may simply result from a decrease in other bacteria and does not equate to an absolute increase in the target bacteria (43). Compared with increases or decreases in single genera, community maturation rate, co-occurrence structure, functional genes, and metabolite profiles may offer stronger biological explanatory power (44). Furthermore, delayed community maturation is not specific to asthma and may also reflect background factors such as prematurity, perinatal antibiotic exposure, infant feeding practices, diet quality, and social environment.

SCFAs, tryptophan metabolites, and bile acids produced by the gut microbiome may serve as candidate mediators through which the gut microbiota influences systemic immunity and airway inflammation (45). However, functional potential cannot be inferred solely from taxonomic composition, and a reduction in potentially SCFAs-producing bacteria does not necessarily imply decreased exposure to bioactive metabolites in the host. Strain function, dietary substrate supply, intestinal absorption, and host metabolism can all influence the final metabolic phenotype (41). Therefore, explanations of changes in the gut microbiome should simultaneously clarify the direction of change for genera or species, functional pathways, and actual metabolite levels, avoiding the use of vague terms like “microbial changes” in place of specific results.

### 2.4 Dynamic changes in the microbiome and asthma phenotypes

The childhood microbiome is not a static structure but changes rapidly early in life and continuously adjusts with age, environmental exposure, infection status, dietary patterns, and medication use (46). Particularly in infancy, the establishment and maturation of microbial communities may participate in shaping immune tolerance, epithelial barriers, and innate immune responses, thereby influencing how children respond to allergens, viral infections, and environmental stimuli (47, 48). Therefore, compared with single time-point sampling results, age-dependent community developmental trajectories may be more informative for elucidating persistent wheezing, asthma development, and differences in clinical phenotypes.

Different asthma phenotypes may correspond to distinct microbiome characteristics. The aforementioned respiratory and gut microbial features may differ between allergic and non-allergic asthma, frequent exacerbation types, and different granulocytic inflammatory phenotypes, but their associations are influenced by age, infection status, sampling time point, and treatment exposure (49). Transient wheezing in infants, virus-induced wheezing, and persistent asthma in school-age children do not represent the same disease process, and samples from acute exacerbation periods and clinically stable periods may also exhibit different community structures (50). Simply combining different age groups, disease states, and phenotypes may obscure truly biologically meaningful subgroup differences.

**Figure 1:**
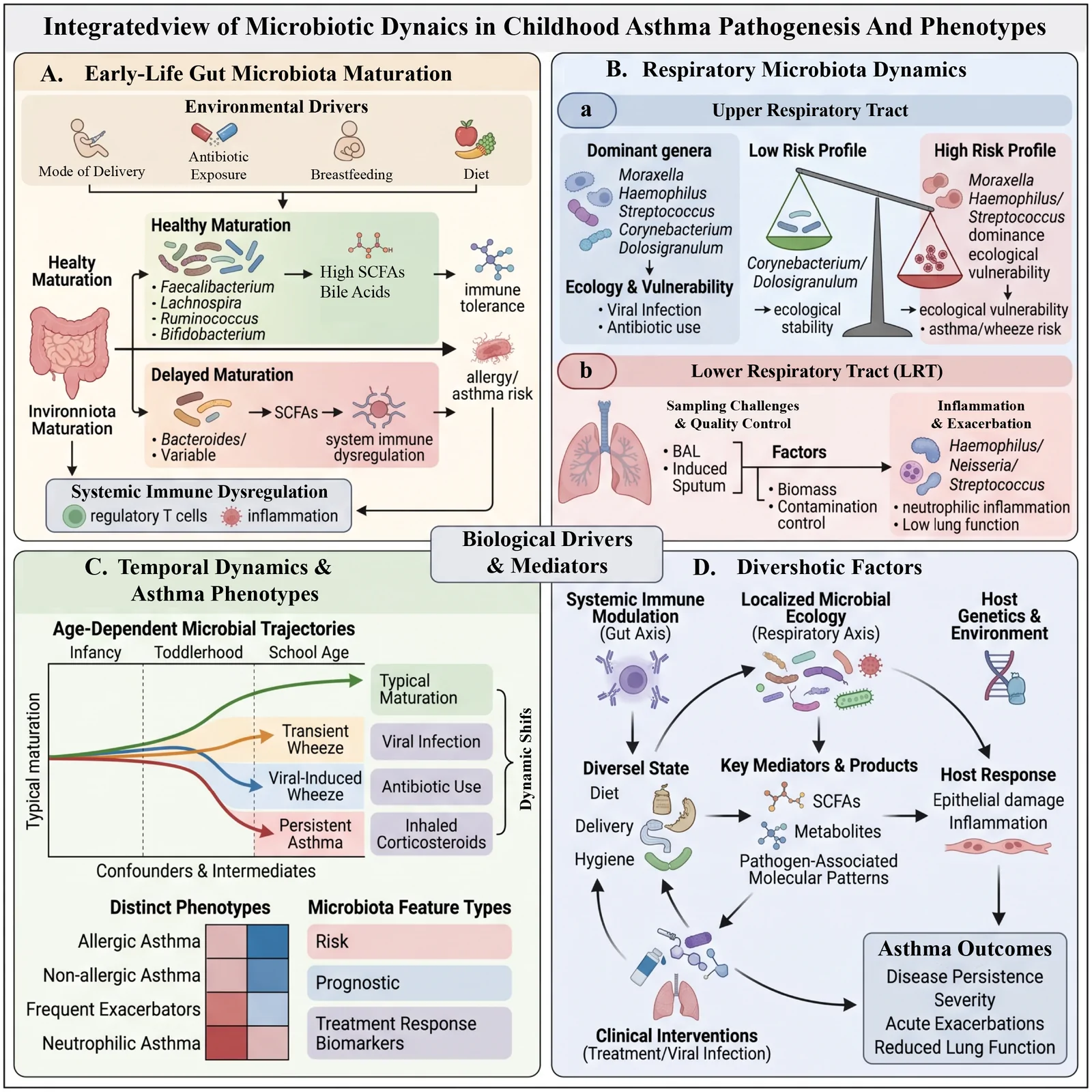
An integrated framework of microbiome dynamic changes, key driving factors, and clinical phenotypes during the development of childhood asthma. **(A)** Maturation of the gut microbiome in early life is influenced by factors such as mode of delivery, antibiotic exposure, breastfeeding, and diet; normal maturation promotes SCFAs production and immune tolerance, while delayed maturation can lead to systemic immune imbalance and increase asthma risk. **(B)** The upper and lower respiratory microbiomes have distinct ecological characteristics and clinical significance; dysbiosis in the upper respiratory microbiota is associated with asthma susceptibility, while changes in the lower respiratory microbiota are associated with increased inflammation and decreased lung function. **(C)** Microbiome maturation trajectories are age-dependent and associated with different asthma phenotypes, disease progression, and potential biomarkers. **(D)** The gut-lung axis, local respiratory microecology, host genetics, and environmental factors collectively influence microbial metabolites and immune responses, ultimately determining the occurrence, severity, acute exacerbations, and lung function outcomes of childhood asthma.

Factors such as viral infections, antibiotic use, and inhaled corticosteroid treatment can further alter microbiome structure and affect its relationship with asthma phenotypes (51). These factors may be either confounding variables or act as mediators in the pathway between microbial changes and clinical outcomes. For example, viral infections can simultaneously trigger acute exacerbations and bacterial community fluctuations, while inhaled corticosteroids reflect disease severity and may also directly reshape the airway microecology (52). Therefore, adjusting for “infection status” or “medication use” at a single time point is usually insufficient to distinguish between disease effects, treatment effects, and microbiome changes themselves. Furthermore, microbial features associated with asthma development may not necessarily predict disease persistence, acute exacerbations, or treatment response. Risk biomarkers, prognostic biomarkers, and treatment response biomarkers have different clinical uses and cannot be substituted for one another simply because they are all associated with asthma (53). Overall, age-dependent community trajectories and their co-variation with infection, treatment, and clinical phenotypes may be more valuable than repeatedly listing specific dominant genera or interpreting abundance differences at a single time point (**Figure 1**).

## 3 Gut–lung axis-mediated microbiota–immune–metabolic mechanisms

The association between the gut microbiome and childhood asthma extends beyond alterations in microbial composition but may also involve microbial metabolic functions and host immune regulation (54). Certain gut microbiota can produce SCFAs, modulate tryptophan and bile acid metabolism, and influence epigenetic modifications and immune cell differentiation (55, 56). Microbial composition, metabolite exposure, and pulmonary immune responses represent interconnected but not equivalent levels of evidence; changes in microbial abundance alone are insufficient to infer their distal biological effects. Therefore, this section focuses on the potential links among microbial metabolic signals, immune regulation, and mucosal barrier function, conceptualizing the gut-lung axis as a bidirectional interaction network rather than a linear causal pathway of “microbial dysbiosis–airway inflammation.”

### 3.1 Microbial metabolites and immune regulation

Microbial metabolites are candidate mediators connecting the microbiome and host immune responses. Among them, SCFAs are the most studied class of metabolites, primarily including acetate, propionate, and butyrate (57, 58). SCFAs can regulate immunity through both receptor-dependent and receptor-independent pathways. Acetate and propionate mainly act on free fatty acid receptors FFAR2 (GPR43) and FFAR3 (GPR41), while butyrate can also activate GPR109A. Expressed in dendritic cells, macrophages, epithelial cells, and select lymphocytes, these receptors regulate chemotaxis, cytokine release, and immune cell activation (59). Intracellularly, butyrate and propionate can inhibit histone deacetylases, increase histone acetylation at relevant gene loci, promote Foxp3 expression and regulatory T cell (Treg) differentiation, and limit excessive dendritic cell maturation and pro-inflammatory cytokine production (60). SCFAs can also alter the glycolytic, fatty acid oxidation, and oxidative phosphorylation states of immune cells, thereby affecting the functional stability of Tregs, effector T cells, and macrophages (61).

In airway allergic responses, these effects may indirectly reduce IgE production driven by IL-4, IL-5, and IL-13, as well as eosinophil recruitment, mucus secretion, and airway hyperresponsiveness, by enhancing Treg-mediated acquired immune tolerance, reducing dendritic cell sensitization to allergens, and suppressing excessive Th2 and ILC2 responses (62). However, the effects of different SCFAs are heterogeneous, and their immunomodulatory roles are influenced by concentration, exposure duration, tissue compartment, and inflammatory context; under different conditions, receptor signaling and cellular metabolic effects may yield different outcomes (63).

Fecal SCFAs levels reflect the net result of intestinal production, absorption, and excretion, which is not equivalent to circulatory exposure and cannot directly represent effective pulmonary concentrations. Their levels are also influenced by dietary fiber intake, strain functionality, intestinal transit, mucosal absorption, and host hepatic metabolism (64). Therefore, the assumption that “reduced potential SCFAs-producing bacteria → decreased fecal SCFAs → impaired pulmonary immune tolerance” is not necessarily valid and requires combined validation through microbial function, metabolite flux, and target cell responses.

In addition to SCFAs, tryptophan metabolites may also participate in mucosal immune regulation. Gut microbes can convert tryptophan into indole, indole-3-aldehyde, indole-3-propionic acid, and other derivatives, some of which can activate the aryl hydrocarbon receptor (AhR) (65, 66). AhR signaling can act on epithelial cells, dendritic cells, T cells, and innate lymphoid cells, promoting IL-22 production, mucus and Antimicrobial peptides expression, and epithelial repair, and participating in the Treg/Th17 balance (67). Thus, reduced tryptophan metabolic capacity may weaken barrier homeostasis and immune tolerance, but its effects are also influenced by dietary tryptophan supply, host metabolic enzyme activity, and the affinity of different AhR ligands (68).

Bile acids undergo co-metabolism by the host and microbes; primary bile acids entering the intestine can be deconjugated and converted into various secondary bile acids by microbes (69). These metabolites can regulate immunity and metabolism through the farnesoid X receptor (FXR) and the membrane receptor TGR5. FXR signaling is involved in bile acid homeostasis, epithelial barrier function, and inflammatory gene expression; TGR5 activation can inhibit NF-κB-mediated pro-inflammatory responses via cAMP-related pathways and affect macrophage and dendritic cell function (70). Some secondary bile acid derivatives can also directly influence Th17 differentiation or promote Treg formation, suggesting their potential involvement in the balance between allergic inflammation and immune tolerance (71).

Additionally, histamine, lipid mediators, and other microbe-derived small molecules may affect mast cell activation, oxidative stress, and airway inflammation (72). However, the robustness of the evidence for different metabolites varies: SCFAs have substantial support from animal and immune mechanism studies, while direct evidence for tryptophan metabolites and secondary bile acids in childhood asthma remains limited, with existing conclusions largely derived from adults, animal models, or other immune diseases (73, 74). Therefore, these metabolites should currently be considered biologically plausible candidate mediators rather than established causal intermediaries in childhood asthma.

### 3.2 Interactive mechanisms of the gut-lung axis

The gut-lung axis emphasizes the bidirectional connection between the gut microbiome, the respiratory microenvironment, and the host immune system (75). Gut-derived metabolites, microbe-associated molecular patterns, and immune signals may influence the lungs via blood circulation, the lymphatic system, or immune cell migration; conversely, pulmonary inflammation and systemic immune changes may affect the gut barrier, metabolic status, and microbial composition (76). Thus, the gut–lung axis should be understood as a dynamic network of metabolic, immune, and barrier signal interactions rather than a unidirectional transmission chain.

At the metabolic signaling level, SCFAs, tryptophan metabolites, and bile acid metabolites, upon entering systemic circulation and maintaining sufficient bioactivity, may act on pulmonary dendritic cells, macrophages, T cells, and innate lymphoid cells (77–79). Among them, SCFAs may support Treg-related immune tolerance and limit excessive Th2 responses, while tryptophan and bile acid metabolites may participate in mucosal immunity and barrier homeostasis through corresponding receptor pathways (80). Their distal effects are not only determined by intestinal production but are also jointly limited by intestinal mucosal absorption, hepatic first-pass metabolism, circulatory transport, and pulmonary target cell receptor expression.

At the immune regulation level, microbe-derived signals may influence the balance among Tregs, Th2, Th17, and innate lymphoid cells (81). Enhanced Treg function helps maintain immune tolerance and limit allergic inflammation, while Th2 and ILC2-related responses can promote IgE production, eosinophilic inflammation, mucus secretion, and airway hyperresponsiveness (82–84). Some atypical or severe asthma phenotypes may also involve Th17 responses and neutrophilic inflammation (85). Alarmins such as TSLP, IL-25, and IL-33 released by the airway epithelium can further amplify signal communication between epithelial cells and immune cells (86). Microbial metabolic signals may influence airway inflammation by altering the activation thresholds of these cells, but they cannot replace direct driving factors such as local allergens, viral infections, and epithelial damage.

At the barrier level, the intestinal and airway epithelia together constitute the mucosal defense system (87). Impaired intestinal barrier function can increase the entry of lipopolysaccharides and other microbe-associated molecular patterns into the circulation, while airway barrier disruption facilitates allergen entry and alarmin release (88, 89). SCFAs, AhR ligands, and bile acid receptor signals may also affect barrier integrity by regulating claudin expression, mucus secretion, antimicrobial peptide production, and epithelial repair (90). Metabolic signals, immune responses, and barrier damage may thus form mutually reinforcing feedback loops.

**Figure 2:**
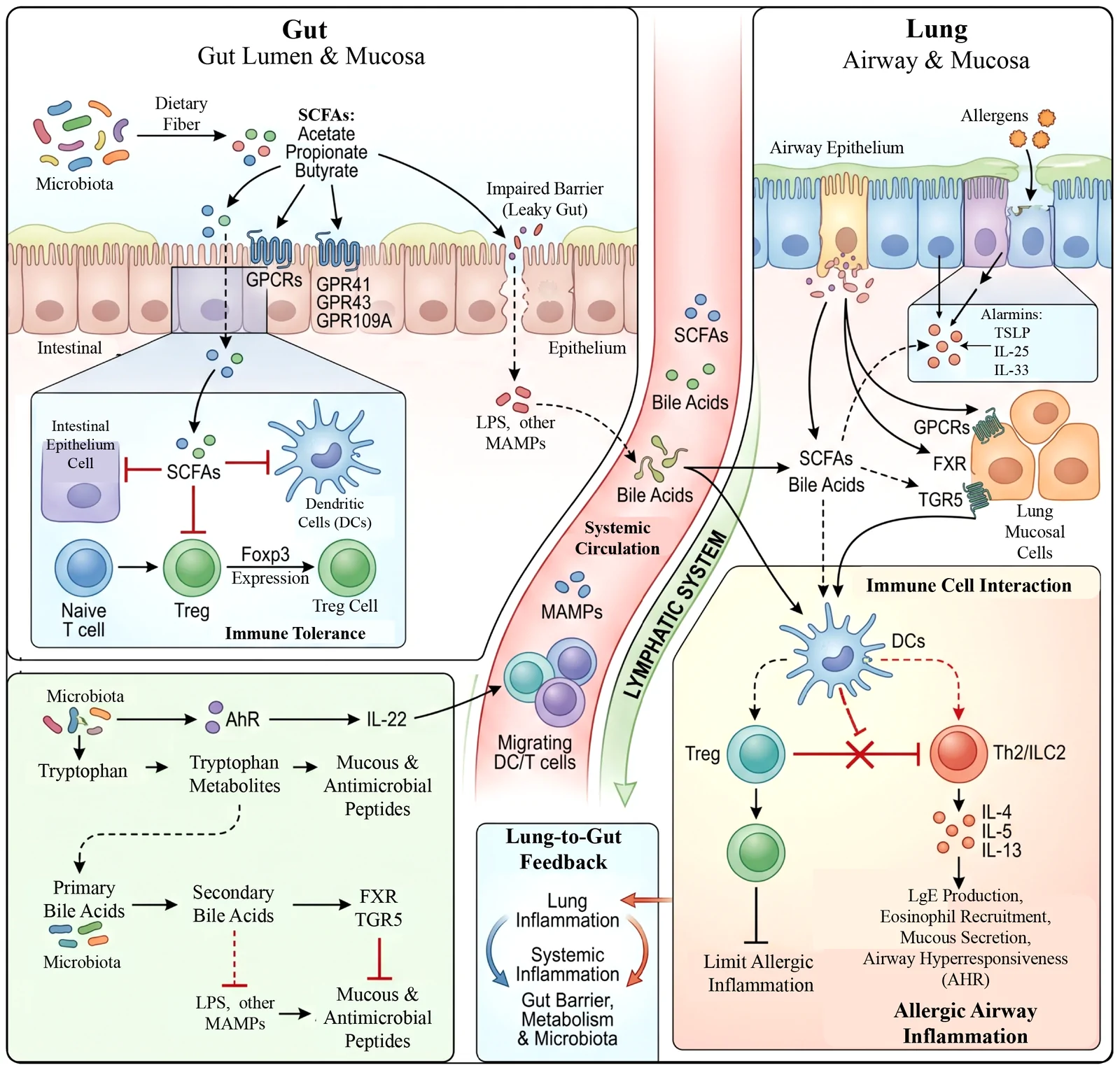
Gut-lung axis-mediated regulation of microbial metabolites, mucosal immunity, and airway inflammation in childhood asthma. Gut microbiota produce SCFAs through dietary fiber fermentation and regulate bile acid and tryptophan metabolites, enhancing intestinal barrier function, promoting Treg differentiation, and maintaining immune tolerance via pathways such as GPCRs, AhR, FXR, and TGR5. When the intestinal barrier is compromised, LPS and MAMPs can enter systemic circulation and, together with SCFAs, bile acids, and migratory immune cells, influence pulmonary mucosal immunity. Allergen stimulation in the lungs induces the release of epithelial alarmins TSLP, IL-25, and IL-33, promoting Th2/ILC2 responses, IgE production, eosinophil recruitment, mucus secretion, and airway hyperresponsiveness. Gut-derived metabolites and Treg responses can limit allergic inflammation, while pulmonary inflammation can reversely affect the gut microbiota through systemic inflammation, barrier, and metabolic changes.

It should be emphasized that detecting metabolites or microbe-associated molecular patterns in the blood does not prove their intestinal origin, effective arrival at the lungs, or ability to achieve concentrations sufficient to alter target cell function. Similarly, changes in the gut microbiota observed after respiratory infections cannot independently demonstrate direct reverse signaling from the lungs to the gut, as confounding factors such as appetite changes, medication use, fever, and systemic inflammation may all contribute. Therefore, the gut-lung axis is currently more suitable as a mechanistic framework integrating microbial metabolism, host immunity, and mucosal barrier function, rather than a validated continuous causal pathway in childhood asthma (**Figure 2**).

## 4 Impact of environmental factors on the childhood microbiome and asthma risk

The early-life microbiome does not develop independently but is continuously shaped by medications, perinatal factors, feeding practices, and living environments. This section focuses on the effects of early antibiotic exposure, delivery and feeding methods, as well as environmental microbes and pollutants, distinguishing between direct effects on asthma risk, microbiome-mediated effects, and associations due to shared background factors.

### 4.1 Impact of early antibiotic exposure

Early antibiotic exposure is one of the important factors affecting the establishment of the gut and respiratory microbiome in children. Early life, especially the first two years after birth, is a critical period for rapid gut microbiota colonization and immune system development (91). Multiple cohort studies and systematic reviews suggest that antibiotic exposure during pregnancy or infancy is associated with an increased risk of subsequent wheezing, asthma, and other allergic diseases in children (92).

Mechanistically, antibiotics may disrupt normal microbial colonization, reduce community diversity and ecological stability, deplete specific commensal taxa, and promote the relative enrichment of opportunistic pathogens, thereby affecting microbial metabolic functions, mucosal barriers, and the establishment of immune tolerance (93). Animal studies have also shown that oral antibiotics can simultaneously alter the gut microbiota, lung microecology, and inflammatory factor expression, providing mechanistic clues for the “gut microbiota perturbation-systemic immune changes—airway inflammation” pathway (94). However, the dosage, exposure duration, and microbiota background in animal experiments differ significantly from clinical usage patterns in children, limiting the direct extrapolation of these findings to confirm that antibiotics increase childhood asthma risk through the microbiome.

Different antibiotic classes, doses, and timing of use may have varying effects on the microbiome. Macrolide antibiotics may have a more persistent impact on gut community structure, while the effects of penicillins, cephalosporins, and other antibiotics also differ (95). Antibiotic exposure during pregnancy may similarly affect the establishment of the neonatal microbiome and be associated with the risk of subsequent allergic diseases (96). However, heterogeneity across studies regarding definitions of exposure windows, antibiotic classes, cumulative doses, infection backgrounds, and asthma outcomes persists, and a consistent dose-response or class-specific relationship has not yet been established (97).

It should be noted that there are at least three competing explanations for the association between antibiotic exposure and asthma risk: first, antibiotics directly disrupt the early microbiome and affect immune development; second, respiratory infections that prompt antibiotic use themselves increase the risk of wheezing and asthma, i.e., confounding by indication; third, children with early wheezing tendencies but not yet diagnosed with asthma are more likely to receive antibiotics due to recurrent respiratory symptoms, leading to reverse causation (98). Infection severity, healthcare-seeking frequency, home environment, and healthcare accessibility may also simultaneously influence antibiotic prescribing and asthma diagnosis (99, 100). Therefore, simply adjusting for the number of infections is usually insufficient to exclude residual confounding from infection type, severity, prescription reason, and early wheezing symptoms. While the rational use of antibiotics and avoiding antimicrobial treatment without confirmed bacterial infection are of clear clinical and public health importance and mitigate unnecessary microbiota disruption, current evidence remains insufficient to conclude that reducing antibiotic use alone can independently prevent childhood asthma.

### 4.2 Delivery mode and feeding method

Delivery mode and feeding method are important early-life factors affecting the establishment of the neonatal microbiome and immune development. Cesarean delivery can alter the initial microbial sources that newborns encounter, resulting in a gut colonization pattern different from that of vaginally delivered children (101, 102). Studies show that colonization by some commensal bacteria may be delayed in cesarean-delivered infants, while the relative abundance of certain opportunistic pathogens is increased, and these changes are potentially associated with an increased risk of subsequent allergic diseases and asthma (103). However, delivery mode is not an isolated exposure. Planned and emergency cesarean deliveries have different clinical indications, and maternal underlying diseases, pregnancy complications, preterm birth, perinatal antibiotic use, and subsequent feeding methods may all confound delivery mode, the childhood microbiome, and respiratory outcomes (104). Therefore, the microbiome may be one of the mediating links through which cesarean delivery affects immune development, or it may simply be a concomitant marker of the combined effects of multiple perinatal factors. Current evidence cannot fully attribute the asthma risk associated with cesarean delivery to initial microbiota differences.

Breast milk contains human milk oligosaccharides, secretory IgA, lactoferrin, and various immunomodulatory components that promote the colonization of commensal bacteria such as Bifidobacterium, thereby facilitating intestinal barrier maturation and the establishment of immune tolerance (105, 106). Some cohort studies and systematic reviews suggest that longer duration of breastfeeding may be associated with a reduced risk of childhood wheezing or asthma (107). Its potential mechanisms may involve promoting microbial community maturation, providing selective microbial substrates, regulating metabolite production, and supporting Treg-related immune tolerance and mucosal barrier development (108). However, the relationship between breastfeeding and childhood asthma is not consistent across all studies. The duration and method of breastfeeding are often correlated with maternal health status, allergy history, socioeconomic status (SES), health behaviors, smoking exposure, and healthcare resources, all of which are independent determinants of asthma risk (109). Furthermore, definitions of exclusive breastfeeding, mixed feeding, and breastfeeding duration vary across studies, complicating the comparison of results. Some studies have not found a clear association between breastfeeding and asthma severity, while household smoking and other environmental exposures may have stronger effects (110). Therefore, breastfeeding should be regarded as an important measure to support infant nutrition, microbiome, and healthy immune system development, but it should not be described as independently preventing childhood asthma through a single microbial mechanism.

### 4.3 Environmental microbial exposure and pollutants

Microbial exposure in the living environment and air pollution may both affect the childhood microbiome, mucosal barrier, and asthma susceptibility (111). With accelerated urbanization, children’s opportunities to interact with natural environments and diverse environmental microbes have decreased, while exposure to indoor and outdoor pollutants, chemicals, and allergens has increased. These factors may jointly participate in the remodeling of the respiratory microecology and immune development (112, 113).

Environmental exposures can influence asthma risk through both direct and indirect pathways. Pollutants can directly induce oxidative stress, damage the airway epithelium, and promote local inflammation, or they may indirectly affect host immunity by altering the respiratory or gut microbial community (114). Conversely, early-life exposure to a certain range of environmental microbes may participate in innate immune training and immune tolerance formation (115). Therefore, environmental factors should not be simply divided into “beneficial microbial exposure” and “harmful pollutant exposure”; their effects depend on the exposure components, dose, time window, and host susceptibility.

Air pollutants such as PM2.5, NO_2_, and SO_2_ are associated with the onset and acute exacerbation of childhood asthma, with potential mechanisms including inducing oxidative stress, damaging the airway epithelial barrier, promoting inflammatory responses, and altering the respiratory microbial community (116, 117). However, it is currently unclear what proportion of the respiratory effects of pollutants is mediated by microbiome changes. Different pollutants often coexist, and indoor chemicals, building materials, allergens, and microbial-derived components may constitute complex mixtures, making it difficult to isolate the independent effect of a single factor (118, 119).

Traditional farm or rural environment exposure is often associated with a lower risk of allergic diseases and asthma, known as the “farm effect” (120, 121). A richer environmental microbiome and related molecules such as lipopolysaccharides and CpG oligonucleotides may exert protective effects by promoting innate immune training, immune tolerance, and the maturation of the gut and respiratory microbiota (122, 123). However, the farm environment simultaneously includes factors such as animal contact, dietary patterns, family size, air composition, and lifestyle, so its protective association may not be entirely mediated by microbial diversity. Therefore, the simplistic assumption that “higher microbial diversity leads to stronger protection” is not valid. The role of environmental microbes is more likely to depend on specific composition, function, exposure intensity, and time window: some microbial signals may promote immune tolerance, while certain bioaerosols, high endotoxin exposure, or pollutant-microbe combinations may also exacerbate airway inflammation (124).

**Figure 3:**
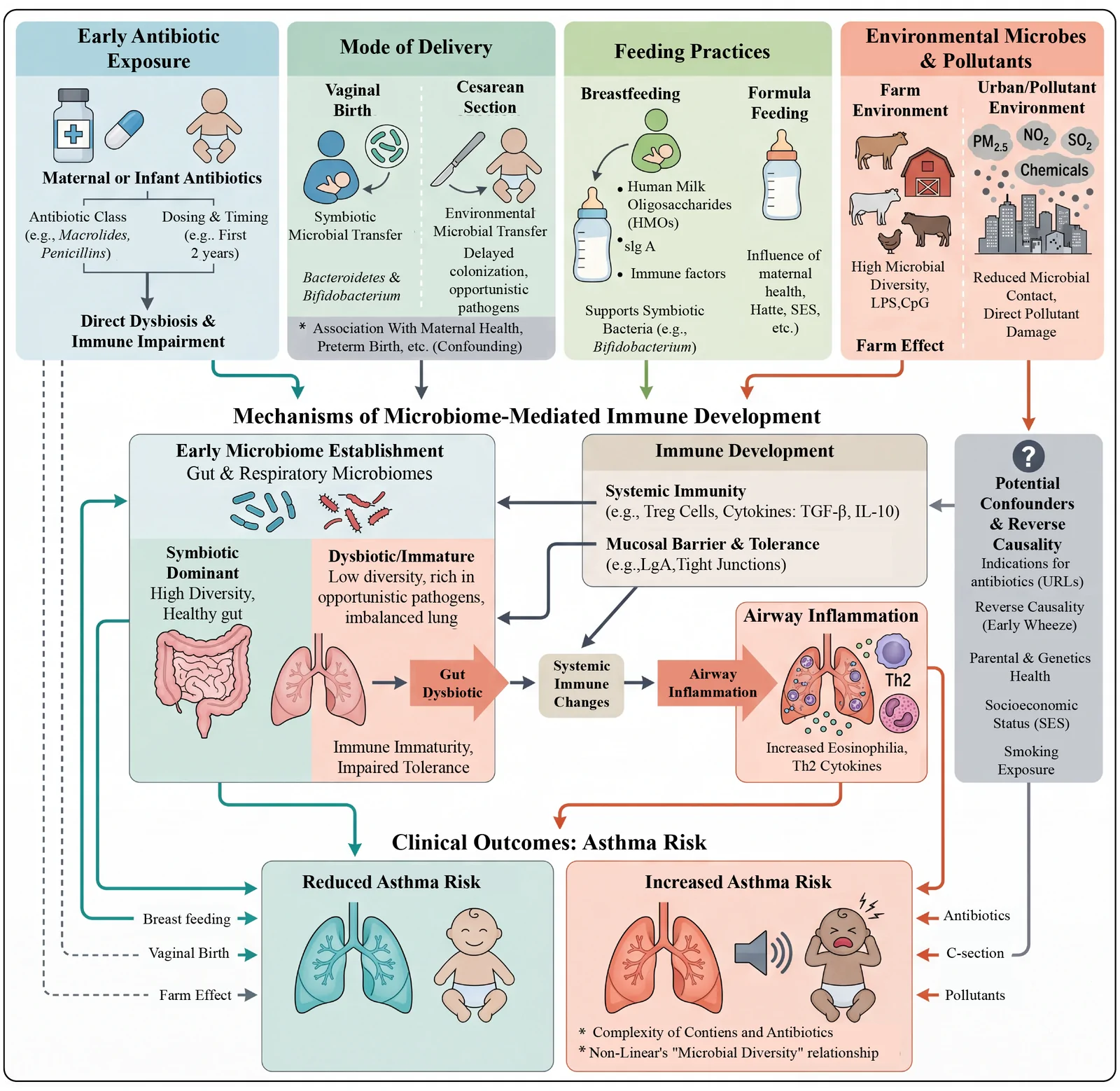
Integrated mechanisms of early-life environmental exposures, microbiome establishment, and childhood asthma risk. This image outlines how early-life antibiotic exposure, delivery mode, feeding pattern, and environmental microbes/pollutants affect the establishment of the gut and respiratory microbiome, and further regulate immune development and asthma risk. Breastfeeding, vaginal delivery, and farm environment exposure can promote commensal bacteria dominance, microbial diversity, and immune tolerance, thereby potentially reducing asthma risk; conversely, early antibiotics, cesarean delivery, formula feeding, and urban pollution exposure can lead to microbiota imbalance, delayed immune maturation, Th2-type airway inflammation, and increased asthma risk. The image also emphasizes confounding factors and reverse causation, such as antibiotic indication, early wheezing, genetic background, socioeconomic status, and smoking exposure, which have important implications for interpreting the microbiome–asthma association.

Overall, environmental factors should be regarded as a complex exposure system composed of microbes, pollutants, lifestyle, and host susceptibility. Future research should integrate individual exposure measurements, environmental microbiome, host immune phenotypes, and longitudinal clinical outcomes to distinguish between microbiome-mediated effects, direct pollutant effects, and their interactions (**Figure 3**).

## 5 Microbiome-based precision prevention strategies

Microbiome-based interventions offer new research directions for the prevention and adjunctive management of childhood asthma, but their mechanistic plausibility, microbiological effects, and clinical benefits belong to different levels of evidence. Changes in microbial composition, metabolites, or inflammatory markers do not necessarily translate into sustained improvements in asthma incidence, acute exacerbations, symptom control, or lung function. Therefore, this section separately evaluates the strategy-specific challenges of different approaches and discusses the identification of responsive populations, intervention matching, and clinical translation boundaries within a biomarker and multi-omics framework.

### 5.1 Probiotic, prebiotic, and synbiotic interventions

Probiotics, prebiotics, and synbiotics are among the most studied microecological modulation strategies. Some randomized controlled trials and systematic reviews suggest that specific preparations may improve outcomes such as lung function, acute exacerbations, inflammatory markers, or quality of life (125). However, positive signals are usually limited to specific strains, specific populations, or certain secondary outcomes. Some studies have observed only changes in microbial composition or inflammatory markers without concurrent improvements in symptom control, acute exacerbations, or lung function, indicating that microbiological responses and clinical benefits need to be evaluated separately (126).

Mechanistically, probiotics may exert effects by modulating gut microbiota, promoting short-chain fatty acid production, enhancing intestinal barrier function, and influencing Treg/Th2 balance and gut–lung axis signaling (59, 127). Strains such as *Lactobacillus* and *Bifidobacterium* have received considerable attention, but different strains exhibit significant differences in colonization ability, metabolic function, and immune effects (128). Single-strain and multi-strain preparations differ in composition, dosage, and mode of action; therefore, they should not be regarded as homogeneous interventions. Multi-strain preparations are not necessarily superior to single-strain ones, as strains may exhibit synergy, competition, or functional antagonism (129).

The key issue with probiotic interventions is not merely whether they are “effective,” but whether the strain, dosage, intervention timing, and host background are matched. Baseline gut microbiota can influence the colonization and functional expression of exogenous strains; age, dietary patterns, prior drug exposure, geographical environment, and asthma immune phenotype may also alter intervention responses (130). For single-strain or multi-strain preparations, viability, colonization duration, functional output, and persistence of effects after discontinuation should be evaluated separately, rather than inferring efficacy solely based on the number of strains in the preparation (131).

Prebiotics and synbiotics can improve gut microecology by promoting the growth of specific commensal bacteria and metabolite production (132). Some studies suggest that prebiotics or synbiotics may have potential preventive or adjunctive management value in high-risk children or specific allergic phenotypes (133). However, intervention effects may be jointly influenced by substrate type, strain combination, dosage, treatment duration, baseline diet, and short-chain fatty acid production capacity. The same preparation may produce different responses under different baseline microbiota and dietary backgrounds, so future comparisons should be stratified by strain, substrate, dosage, intervention window, and target population, rather than merely evaluating overall average effects.

### 5.2 Nutritional interventions and lifestyle adjustments

High-fiber diets can promote the production of SCFAs by gut microbiota, potentially supporting immune tolerance and mucosal barrier stability (134); dietary patterns rich in fruits, vegetables, whole grains, and unsaturated fatty acids may also be associated with lower inflammatory levels and better respiratory health (135). Conversely, high-fat, low-fiber, and ultra-processed food intake may disrupt microecological stability and create a pro-inflammatory metabolic environment (136).

The research focus of nutritional interventions is gradually shifting from uniform dietary recommendations to precision nutrition based on metabolic and microbial characteristics. Children with insufficient baseline dietary fiber intake or low short-chain fatty acid production capacity may theoretically be more suitable candidates for high-fiber diets or targeted prebiotic interventions (137); those with obesity, insulin resistance, or other metabolic abnormalities may require simultaneous attention to energy balance, weight control, and metabolic inflammation. Different allergic statuses and granulocytic inflammatory phenotypes may also correspond to different nutritional intervention goals (138).

Fecal or circulating SCFAs, tryptophan metabolites, bile acids, and inflammatory markers can serve as candidate response biomarkers for assessing microbial and metabolic changes following dietary interventions (139). However, changes in a single metabolite are insufficient to demonstrate clinical benefit, and nutritional studies should simultaneously evaluate actual dietary intake, microbial function, metabolites, body weight and metabolic status, as well as clinical outcomes such as symptom control, acute exacerbations, and lung function.

Major methodological limitations of nutritional interventions include the difficulty of adequate blinding in dietary trials, challenges in accurately measuring actual intake, and difficulty in maintaining long-term adherence (140). Dietary patterns are also closely related to family socioeconomic status, parental health awareness, body weight, physical activity, smoking exposure, and healthcare resources, so observed respiratory health benefits may not be entirely attributable to microbiome changes (141). Even if dietary interventions are accompanied by changes in microbiota or SCFAs, it is necessary to further distinguish between direct metabolic effects of diet and microbiome-mediated effects.

Human milk oligosaccharides, secretory IgA, lactoferrin, and other immunologically active components in breast milk can promote the colonization of commensal bacteria such as Bifidobacterium and support the intestinal barrier and immune development (142). Some cohort studies suggest that breastfeeding may be associated with a lower risk of wheezing, respiratory infections, or asthma, but this association is also influenced by maternal allergic status, home environment, air pollution, socioeconomic factors, and genetic background (143, 144). However, breastfeeding is a physiological nutritional process with complex components and exposure patterns, rather than a standardized microecological intervention with uniform composition, dosage, and duration. Its association with childhood asthma risk is also affected by maternal health, family environment, and socioeconomic factors, so its protective effects should not be attributed to a single microbial mechanism.

Lifestyle adjustments constitute an important component of overall nutritional and environmental management. Reducing ultra-processed food intake, improving dietary quality, and controlling obesity and metabolic abnormalities may jointly influence the microbiome, host metabolism, and airway inflammation; environmental factors such as antibiotics, pollution, and secondhand smoke can further alter intervention responses (145, 146). Since these factors often coexist, their independent effects and the extent of microbiome mediation are difficult to fully separate. Precision nutrition prioritizes tailoring intervention plans to baseline dietary intake, microbial function, metabolic status, and asthma endotypes. Current major obstacles include insufficient dietary adherence, measurement errors in actual intake, residual confounding, lack of unified stratification criteria, and absence of direct evidence showing that biomarker-guided diets are superior to general healthy dietary recommendations.

### 5.3 Fecal microbiota transplantation and novel microecological therapies: exploratory directions

Fecal microbiota transplantation (FMT) is a microecological intervention that modulates host immune and metabolic status by reshaping the gut microbial community (147). Based on the gut-lung axis theory, FMT may influence airway inflammation by altering gut microbial composition, restoring certain microbial functions, and modulating metabolic and immune signals (148). Animal studies have shown that FMT or microbial remodeling can alleviate some allergic airway inflammation and immune imbalance, providing clues to its potential mechanisms (149).

The core issue with FMT lies in the complexity and difficulty of fully defining its input components. There is no unified standard for “healthy donor microbiota,” as the pediatric microbiome changes dynamically with age, geography, diet, and living environment; ecological compatibility between donor and recipient may also affect colonization and functional recovery (150). Therefore, microbial remodeling effects observed in animal models cannot be directly extrapolated to donor selection, intervention dosage, or duration in pediatric patients.

The microbiome and immune system of children are still in a developmental stage, and FMT involves issues such as donor screening, infection transmission, antimicrobial resistance gene transfer, long-term ecological changes, ethical review, and regulation (151). A single microbial remodeling may produce only transient changes or may lead to unpredictable long-term colonization and metabolic consequences. Beyond known pathogens, the donor microbiota may inadvertently co-transfer unrecognized viruses, opportunistic pathogens, resistance genes, and microbial components with potential metabolic activity. Whether the long-term ecological consequences are reversible and whether there are specific risks during childhood development remain priority issues that FMT research must address (152).

Well-defined strain combinations, postbiotics, microbe-derived metabolites, targeted prebiotics, and multi-omics-guided personalized interventions may offer superior component control and facilitate quality standardization compared to traditional FMT (153). Rational design strategies encompass: designing defined microbial consortia based on missing commensal bacteria or impaired functional pathways; selecting postbiotics or metabolite supplementation based on deficiencies in SCFAs or other microbe-derived metabolites; and determining intervention targets based on immune endotypes such as Th2, Th17, neutrophilic inflammation, or immune tolerance abnormalities (154).

Microbial and metabolic markers can also be used to monitor target strain colonization, functional pathway recovery, metabolite changes, and potential adverse reactions. Evaluation indicators should include absolute abundance of target strains, functional genes, fecal or circulating metabolites, immune responses, and clinical outcomes, rather than relying solely on short-term relative abundance changes to assess therapeutic efficacy. For such novel therapies, it is still necessary to clarify optimal dosage, applicable age, colonization stability, long-term ecological safety, as well as quality control and regulatory requirements.

### 5.4 Biomarker-guided multi-omics stratification and personalized intervention

The core of precision prevention is to identify children with specific risk characteristics or potential treatment responses, rather than applying a uniform microecological intervention to all patients. Candidate biomarkers for childhood asthma can be used for risk prediction, phenotype or endotype stratification, prognosis assessment, and treatment response prediction, including early-life microbial maturity, respiratory microbiota subtypes, microbial metabolites, and immune phenotypes (73). Different types of biomarkers serve distinct purposes: risk markers identify high-risk children, predictive markers select potential beneficiaries, response markers monitor intervention effects, and safety markers identify adverse ecological or immune changes; these functions are not interchangeable (155).

Given the limited cross-population stability of single bacterial genera, multi-omics integration may improve risk stratification and mechanistic interpretation. Metagenomics, metabolomics, transcriptomics, proteomics, and immune phenotypes can be analyzed together with age, lung function, allergic status, diet, medication, and environmental exposure to identify more biologically meaningful asthma endotypes. However, increased model complexity does not necessarily lead to higher clinical value. Batch effects, insufficient sample size, excessive features, overfitting in small samples, poor algorithm interpretability, detection costs, and a lack of cross-regional reproducibility may all reduce model generalizability (156).

**Figure 4:**
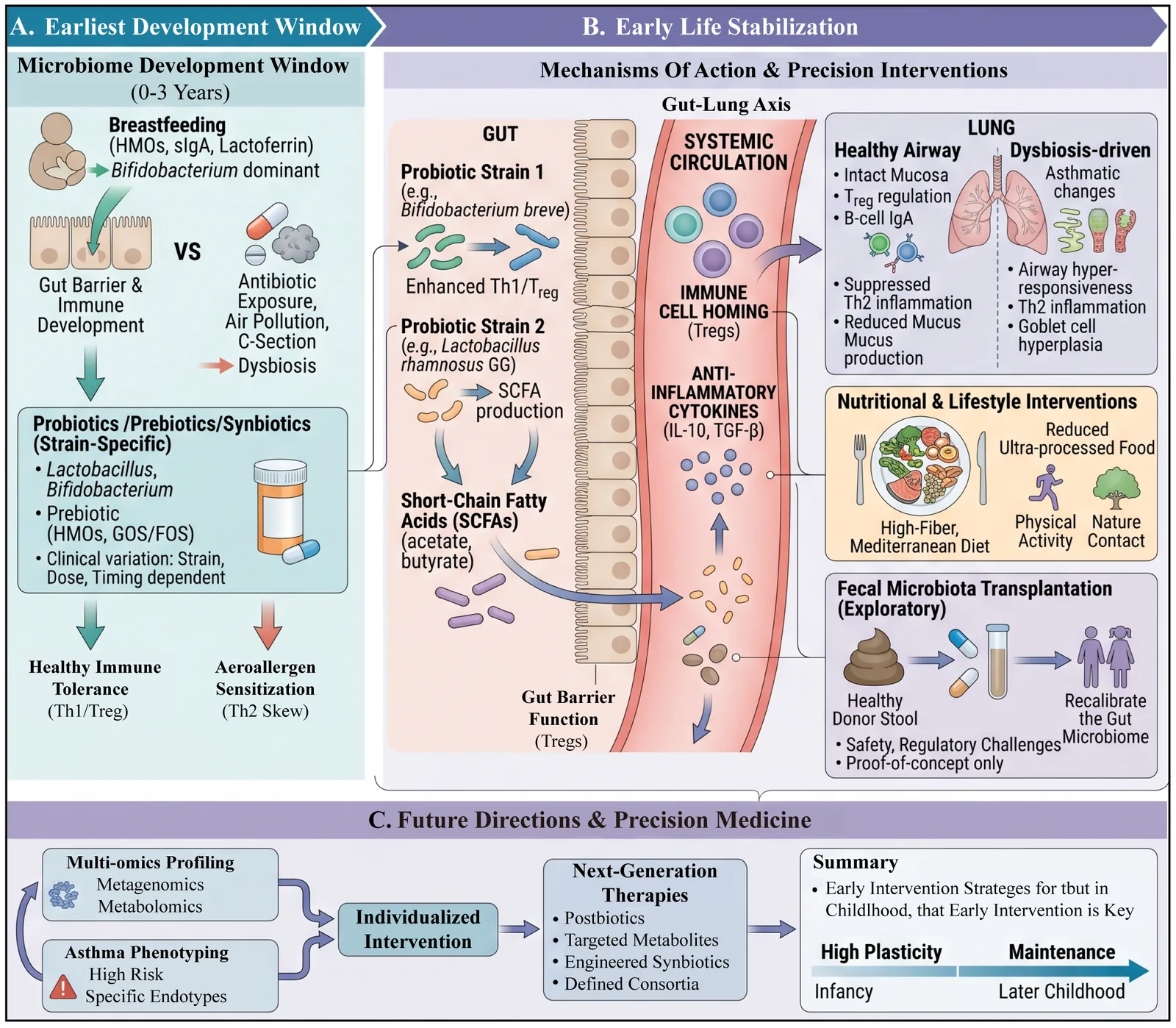
Early-life microbiome intervention windows, gut–lung axis mechanisms, and precision prevention strategies for childhood asthma. This figure outlines the early-life microbiome development window and its relationship with childhood asthma risk. **(A)** The period from 0 to 3 years is a highly plastic stage for the microbiome and immune system; breastfeeding can promote Bifidobacterium dominance, intestinal barrier maturation, and immune tolerance through HMOs, sIgA, and lactoferrin; while antibiotics, air pollution, and cesarean section may induce microbial imbalance and Th2 bias. **(B)** Specific probiotics, prebiotics, and synbiotics can modulate Th1/Treg balance, promote short-chain fatty acid production, enhance intestinal barrier function, and influence lung immune homing, anti-inflammatory factor release, and airway inflammation through systemic circulation. Nutrition, lifestyle, and exploratory fecal microbiota transplantation can also serve as adjunctive intervention directions. **(C)** Future precision prevention should integrate multi-omics analysis, asthma phenotype stratification, and personalized intervention, developing next-generation strategies such as postbiotics, targeted metabolites, engineered synbiotics, and defined microbial consortia.

Theoretical personalized pathways include clinical and environmental assessment, microbiome and metabolite testing, multi-omics subtyping, identification of actionable pathways, intervention matching, and efficacy and safety monitoring. For example, individuals with insufficient short-chain fatty acid production capacity may benefit from high-fiber diets or serve as subjects for targeted prebiotic trials. However, there is currently insufficient evidence to suggest that intervention selection based on microbial markers is superior to traditional clinical stratification (157) (**Figure 4**). Furthermore, protocols for sample collection, storage, sequencing, and analysis lack standardization. Moreover, the dynamic nature of the pediatric microbiome across ages renders single measurements potentially unrepresentative. Finally, existing models are predominantly derived from single-center, small-sample studies and lack both cross-cohort validation and biomarker-guided randomized trials in children. Therefore, biomarker- and multi-omics-guided personalized interventions remain in the research stage, and their clinical application requires further standardization, external validation, and prospective utility evaluation (**Table 1**).

**Table 1:** Main directions and evidence boundaries of microbiome-related precision prevention strategies in childhood asthma.

Intervention category	Representative strategies/ applicable scenarios	Main mechanisms	Potential value	Evidence boundaries and current limitations	Future optimization directions	Reference
Probiotic intervention	Specific strains such as *Lactobacillus, Bifidobacterium;* stratified by age, allergic phenotype, and baseline microbiota.	Regulate gut microbiota, promote SCFA production, enhance intestinal barrier function, influence Treg/Th2 balance and gut–lung axis signaling.	May improve inflammatory indicators, symptom control, acute attacks, or quality of life in some children.	Efficacy is strain-, dose-, timing-, and host-dependent; microbiota changes do not equate to clinical benefit.	Evaluate strain viability, colonization duration, functional output, and maintenance of effects after discontinuation separately.	(59, 125–131)
Prebiotic intervention	GOS/FOS, inulin, HMOs-like substrates; suitable for children with insufficient dietary fiber or low SCFAs-producing function.	Promote commensal bacterial growth, increase production of metabolites such as SCFAs, improve barrier and immune tolerance.	May have preventive or adjunctive management potential in high-risk children or specific allergic phenotypes.	Affected by substrate type, dose, course, baseline diet, and microbiota function; protocols are inconsistent.	Conduct stratified comparisons by substrate, dose, intervention window, baseline diet, and microbiota function.	(132, 133)
Synbiotic intervention	Combined formulations of probiotics and prebiotics.	Promote colonization and metabolic functional output of target strains, enhance immunomodulation.	May improve the microecological regulation effect in specific populations.	Strain-substrate combinations are complex; synergy, competition, or functional cancellation may occur; standardization is insufficient.	Clarify strain-substrate matching principles, establish standards for composition, dosage, and course.	(129, 132, 133)
Precision nutritional intervention	High-fiber diet, Mediterranean diet, reduction of ultra-processed foods; combined with stratification by obesity, metabolic abnormalities, and allergic endotypes.	Promote SCFAs production, improve metabolic inflammation, support mucosal barrier and immune tolerance.	Relatively high safety; suitable as a foundational strategy for long-term prevention and adjunctive management.	Dietary adherence, intake assessment, and blinded implementation are difficult; need to distinguish direct effects from microbiota-mediated effects.	Simultaneously evaluate dietary intake, microbiota function, metabolites, body weight, inflammation, and asthma outcomes.	(137–141)
Breastfeeding support	HMOs, sIgA, lactoferrin, and other bioactive components of breast milk.	Promote Bifidobacterium colonization, support intestinal barrier maturation and early immune development.	Helps establish the infant microbiome and immune homeostasis; may reduce the risk of wheezing or respiratory infections.	This is a complex physiological nutritional exposure, not a standardized microecological intervention; influenced by maternal, genetic, environmental, and social factors.	Avoid attributing protective effects to a single microbial mechanism; integrate maternal, infant, and environmental factors for comprehensive analysis.	(142–144)
Lifestyle and environmental management	Reduce unnecessary antibiotics, lower pollution and secondhand smoke exposure, improve dietary quality, control obesity.	Jointly affect the microbiome, host metabolism, and airway inflammation.	Can serve as important background measures for childhood asthma prevention and adjunctive management.	Multiple factors coexist; independent effects and the proportion mediated by the microbiome are difficult to isolate; high-quality long-term evidence is insufficient.	Use long-term follow-up and multifactorial models to distinguish environmental, metabolic, and microbiome effects.	(145, 146)
Fecal microbiota transplantation (FMT)	Donor microbiota reconstruction; currently mainly limited to mechanistic exploration.	Remodel gut microbiota, alter metabolite profiles and systemic immune responses.	Animal studies suggest potential to reduce allergic airway inflammation.	Still highly exploratory in childhood asthma; concerns include infection transmission, resistance gene transfer, long-term ecological impact, ethical and regulatory issues.	Prioritize donor screening, ecological safety, long-term reversibility, and developmental stage-specific risks in children.	(148–152)
Novel microecological therapies	Defined strain consortia, postbiotics, microbial metabolite supplementation, targeted prebiotics.	Aim to precisely restore missing microbiota, modulate impaired functional pathways, or target specific immune endotypes.	Components are more clearly defined, making them theoretically easier to standardize and subject to quality control compared to FMT.	Optimal dose, applicable age, colonization stability, long-term safety, and clinical benefit are not yet established.	Establish systems for component definition, potency testing, quality control, and long-term safety monitoring.	(153, 154)
Biomarker- guided stratification	Early-life microbiota maturity, respiratory microbiota subtypes, SCFAs, tryptophan metabolites, bile acids, immune phenotypes.	Used for risk prediction, endotype stratification, efficacy monitoring, and safety assessment.	Helps identify potentially responsive populations and match intervention strategies.	Different biomarker uses should not be conflated; the stability of a single genus or single metabolite may be limited.	Distinguish risk, predictive, response, and safety biomarkers; conduct repeated testing and external validation.	(73, 155)
Multi-omics individualized intervention	Integration of metagenomics, metabolomics, transcriptomics, proteomics, immune phenotypes, and clinical environmental factors.	Construct more stable models of asthma risk and treatment response.	Holds promise for advancing “population stratification-intervention matching-dynamic monitoring.”	Challenges include insufficient process standardization, batch effects, overfitting, high costs, poor cross-cohort reproducibility, and a lack of randomized trials in children.	Conduct cross-cohort validation and biomarker-guided prospective randomized trials to demonstrate superiority over traditional clinical stratification.	(156, 157)

## 6 Microbiome and atopic progression: expanding perspectives

### 6.1 The concept of atopic progression and its association with the microbiome

The “atopic march” is commonly used to describe the sequential occurrence of atopic diseases in some children, where atopic dermatitis (AD) or food allergy first appears in infancy, followed by respiratory allergic diseases such as allergic asthma and allergic rhinitis (158, 159). This concept suggests that early skin barrier abnormalities, Th2-type inflammatory tendencies, environmental exposures, and immune developmental status may jointly influence the risk of subsequent respiratory allergic diseases (160, 161). However, the atopic march does not follow a fixed sequence of “atopic dermatitis-food allergy-asthma or allergic rhinitis” in all affected children. While some children present with only a single atopic disease, others directly develop respiratory allergies, and a further subset experiences remission of early skin or food allergy symptoms with age without progressing to asthma (162). Therefore, the atopic march is more accurately conceptualized as several possible clinical trajectories rather than a linear process in which one disease inevitably progresses to another.

Another interpretation is that different atopic diseases may share genetic susceptibility, epithelial barrier defects, Th2-type immune bias, and environmental exposures, manifesting as different organ phenotypes (160). Within this framework, the association between early AD and subsequent asthma may reflect either a biological link between diseases or a common risk background, and the sequence of occurrence alone cannot prove that the former directly causes the latter.

From a microbiome perspective, children with AD often exhibit reduced skin microbial diversity and predominant colonization with Staphylococcus aureus, which may be associated with amplification of local inflammation, barrier damage, and systemic immune sensitization (163, 164). Abnormalities in the gut microbiome may also affect the establishment of immune tolerance and participate in the regulation of Treg/Th2 balance and allergic phenotype shaping through metabolites such as SCFAs (165). However, current evidence is insufficient to regard the microbiome as an independent driver that sequentially promotes atopic diseases. A more plausible explanation is that the skin and gut microbiome, together with the epithelial barrier, host immunity, and environmental exposures, alter the likelihood of onset, combination patterns, and clinical trajectories of different atopic phenotypes.

### 6.2 Interactions of the skin-gut-lung microbial axis

**Figure 5:**
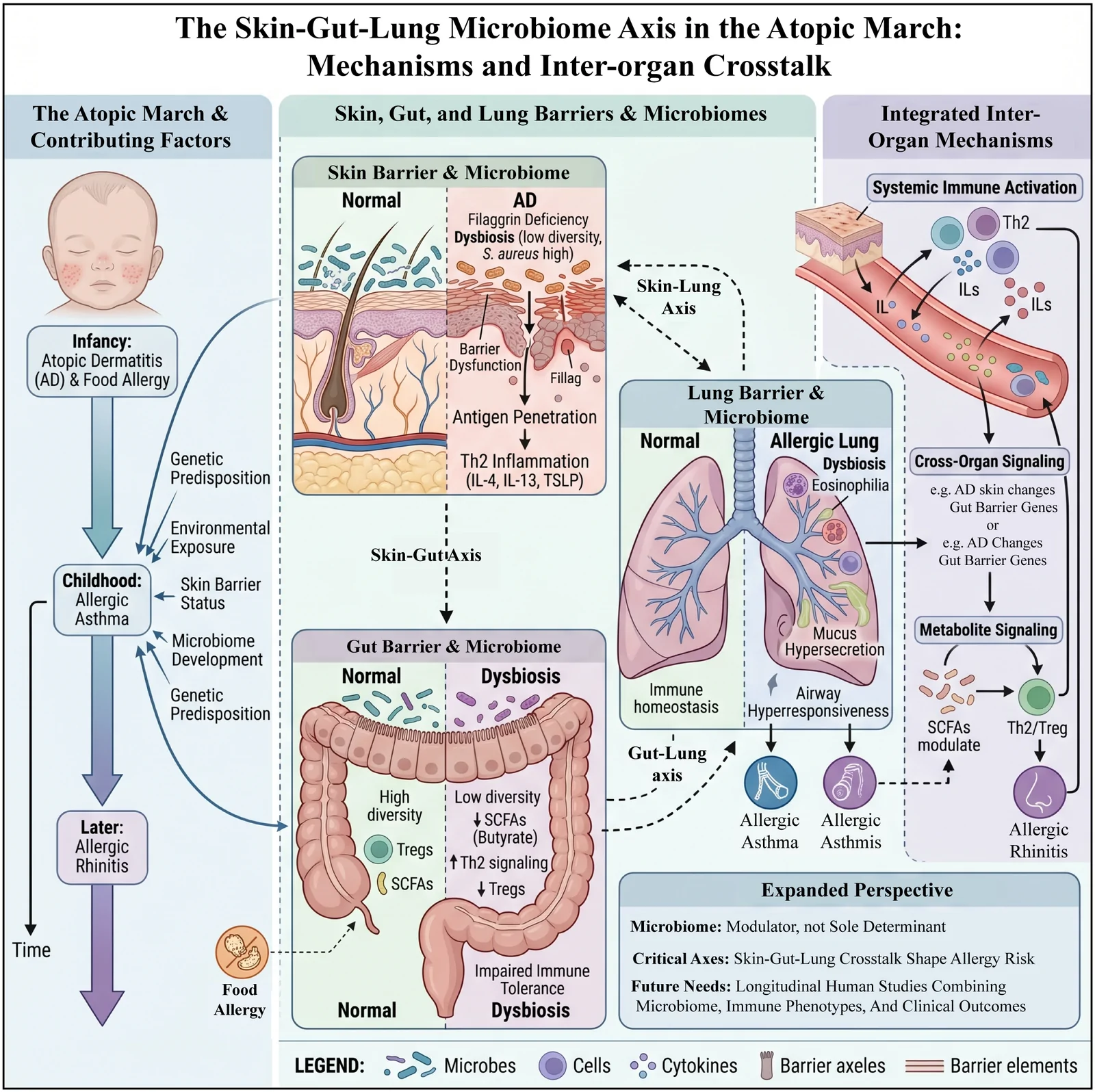
Mechanisms of inter-organ interactions in the “skin–gut–lung” microbial axis during atopic progression. This figure outlines the interactions between the skin, gut, and lung barriers and their microbiota during atopic progression. Skin barrier damage and gut microbiota imbalance can affect lung immune homeostasis through systemic immune activation, metabolite signaling, and Th2/Treg dysregulation, promoting phenotypes such as asthma and allergic rhinitis; conversely, healthy skin and gut microecology help maintain immune tolerance and reduce the risk of allergic disease progression.

The skin, gut, and respiratory tract constitute critical barrier tissues, and their microbial communities may establish cross-organ crosstalk through immune signals, metabolites, and inflammatory mediators (166). The “skin-gut-lung microbial axis” has thus been proposed to elucidate the potential microecological and immune associations among AD, food allergy, asthma, and allergic rhinitis. Animal studies provide mechanistic clues for this framework (167). For example, skin barrier damage in mice can be accompanied by changes in gut microbiota composition and intestinal barrier-related gene expression, suggesting that skin inflammation may affect the distal gut microbiome (168). Additionally, gut microbiota and metabolites such as SCFAs may influence airway inflammation and allergic susceptibility through systemic immune regulation (169). These findings indicate that local barrier damage and microecological changes may exert cross-organ effects via circulating metabolites, immune cells, and inflammatory signals. However, parallel changes in the skin, gut, and respiratory tract may also arise from shared genetic, immune, and environmental backgrounds, rather than necessarily representing a sequential transmission of microecological abnormalities from one organ to drive pathology in another. Therefore, the “skin–gut–lung axis” should not be reduced to a unidirectional linear pathway from the skin to the gut and then to the lungs. Thus, the skin–gut–lung axis currently serves primarily as an integrative cross-organ explanatory framework linking barrier tissues, the microbiome, and host immunity, and its temporal sequence and necessary intermediate steps still require validation through longitudinal human studies (**Figure 5**).

## 7 Current controversies, evidence gaps, and priority research questions

### 7.1 Causal inference and reproducibility of biomarkers

Research on the microbiome in childhood asthma is primarily constrained by unclear causal direction. Most existing studies collect samples after wheezing, asthma diagnosis, or acute exacerbation, making it difficult to distinguish whether microbiome alterations are disease drivers, mediating factors, or secondary changes caused by infection, inflammation, and treatment. Diet, medication, disease severity, and living environment can also simultaneously affect both the microbiome and asthma outcomes; therefore, correlations cannot directly establish independent causal effects. The direction and effect size of associations for multiple candidate respiratory and gut microbial biomarkers have been inconsistent across studies. Age, geographic region, diet, infection status, asthma phenotype, sampling site, and detection methods may all influence results. Particularly in low-biomass respiratory samples, oropharyngeal carryover and reagent contamination may interfere with true signals; relative abundance and genus-level analyses also fail to reflect absolute microbial load and strain-level function. Therefore, future research should adopt standardized longitudinal repeated sampling, contamination control, and absolute quantification, combined with strain identification, functional omics, and comprehensive exposure records. Candidate biomarkers also require independent external validation and must demonstrate incremental predictive value over traditional clinical indicators.

### 7.2 Gut-lung axis evidence chain and asthma heterogeneity

The gut-lung axis provides an important framework for explaining the link between gut microbiota and pulmonary immunity, but a complete evidence chain in children has not yet been established. Longitudinal data spanning “gut microbial function-circulating metabolic signals-pulmonary immune responses-clinical outcomes” remain lacking, and fecal microbiota or metabolites cannot directly represent effective pulmonary exposure. Animal models support the biological plausibility of gut-lung crosstalk, but their immune development, allergen exposure, and disease processes diverge significantly from those observed in childhood asthma and cannot directly extrapolate transmission pathways or effect sizes to children. Therefore, the gut–lung axis currently remains a candidate mechanistic framework rather than a validated continuous causal pathway. Childhood asthma also exhibits significant age and phenotype-related heterogeneity. Transient wheezing, viral-associated wheezing, and persistent asthma, as well as different atopic statuses and inflammatory endotypes, may correspond to distinct microbial signatures and intervention windows. Future studies should stratify by age and endotype, and simultaneously analyze gut, circulating, and respiratory signals to clarify the temporal sequence and target populations of cross-organ effects.

### 7.3 Clinical translation boundaries of microbiome-based interventions

Strategies such as probiotics, prebiotics, nutritional interventions, defined microbial consortia, and FMT have mechanistic plausibility, but microbiological changes do not necessarily translate into improvements in asthma incidence, acute exacerbations, lung function, or long-term disease trajectories. Primary prevention, adjunctive management, and disease-modifying therapy involve different populations, intervention windows, and clinical endpoints, and are not interchangeable based on short-term biomarker changes. Future trials should adopt standardized preparations and utilize clinically meaningful outcomes, and evaluate colonization persistence, post-discontinuation effects, and long-term safety. For potent microbiota-remodeling strategies such as FMT, rigorous monitoring is required for infection transmission, antimicrobial resistance gene transfer, and long-term ecological and metabolic consequences. Biomarkers and multi-omics models may support patient stratification, intervention matching, and treatment monitoring, but unified thresholds, cross-cohort validation, and prospective evaluation of clinical utility are currently lacking. Research priorities should shift from the continuous identification of differential microbiota to the validation of biomarkers with clear temporal ordering, reproducible functional effects, and incremental clinical value, as well as the completion of the clinical translation evidence chain through biomarker-guided randomized trials and long-term follow-up (**Table 2**).

**Table 2:** Key controversies and priority directions in microbiome research on childhood asthma.

Research topic	Core controversy	Key gaps	Priority research questions
Causality and Biomarker Stability	Whether microbial dysbiosis is a cause, consequence, or mediator remains unclear.	Cross-sectional studies are predominant, with insufficient control of confounders and limited reproducibility of candidate biomarkers.	Establish longitudinal cohorts, implement standardized sampling, absolute quantification, contamination control, and external validation.
Gut–Lung Axis and Asthma	Heterogeneity Consistent evidence is lacking on whether gut signals truly influence childhood airway immunity.	Absence of synchronized data on gut, circulation, respiratory, and clinical outcomes.	Stratify by age, wheezing phenotype, allergic status, and inflammatory endotype to clarify the sequence of action.
Microbiome Intervention	Translation Alterations in microbiota or metabolites do not necessarily translate into clinical benefit.	Inconsistent formulations, doses, intervention windows, and endpoints; insufficient long-term safety evidence.	Conduct biomarker-guided randomized trials to evaluate acute exacerbations, lung function, and long-term disease trajectory.
Multi-omics Precision Prevention	Whether complex models can enhance clinical decision-making value remains to be validated.	Inconsistent thresholds, insufficient cross-cohort validation, overfitting, and cost issues.	Validate biomarkers with incremental predictive value and construct interpretable, generalizable stratification models.

## 8 Conclusion

Existing evidence consistently indicates that the childhood microbiome exhibits significant age- and environment-dependent characteristics, and is continuously shaped by factors such as infection, diet, medication, and living environment. Repeated associations have been observed between certain respiratory microbiota features and asthma exacerbation, airway inflammation, and abnormal lung function, and early-life exposure may also influence the developmental trajectory of gut and respiratory microbial communities. Therefore, age-dependent community changes, functional pathways, and metabolic characteristics may warrant greater research attention than the abundance of specific genera at a single time point. However, the causal direction between microbiome alterations and childhood asthma remains unclear, and the comprehensive mechanistic link of “gut microbial function-circulating signals-pulmonary immune responses-clinical outcomes” within the gut–lung axis has not yet been established. The reproducibility of candidate microbial features across regions and populations also requires further validation. Microbiome abnormalities may either contribute to disease pathogenesis or result from infection, inflammation, treatment, or shared environmental exposures. At present, the microbiome and its metabolites cannot be independently used for the diagnosis, phenotyping, or treatment decisions of childhood asthma. Multi-omics integration and biomarkers guide patient stratification, intervention selection, and therapeutic monitoring, offering potential pathways for individualized management, but their incremental clinical value has not been fully demonstrated. Probiotics, prebiotics, nutritional interventions, and other microbial community-based strategies may serve as research-oriented or adjunctive management approaches, but they cannot replace standard treatment. Overall, microbiome research is transitioning from descriptive community associations toward functional validation and precise stratification, yet its integration into routine clinical practice for childhood asthma still requires more robust evidence of causality and clinical utility.

## References

[1] Wolters AAB Kersten ETG Koppelman GH . Genetics of preschool wheeze and its progression to childhood asthma. Pediatr Allergy Immunol. (2024) 35:e14067. doi: 10.1111/pai.14067 38284918

[2] Pijnenburg MW Frey U De Jongste JC Saglani S . Childhood asthma: pathogenesis and phenotypes. Eur Respir J. (2022) 59(6):2100731. doi: 10.1183/13993003.00731-2021 34711541

[3] Yu J Jang HB Lee HJ Kim YY . Lessons from Korean childhood asthma study cohort and needs for long-term prospective study. Jugan Geongang Gwa Jilbyeong. (2023) 16:867–79. doi: 10.56786/phwr.2023.16.26.4 41332449 PMC12480151

[4] Conrad LA Cabana MD Rastogi D . Defining pediatric asthma: phenotypes to endotypes and beyond. Pediatr Res. (2021) 90:45–51. doi: 10.1038/s41390-020-01231-6 33173175 PMC8107196

[5] Hossenbaccus L Linton S Ramchandani R Gallant MJ Ellis AK . Insights into allergic risk factors from birth cohort studies. Ann Allergy Asthma Immunol. (2021) 127:312–7. doi: 10.1016/j.anai.2021.04.025 33971362

[6] Carter MJ Carrol ED Ranjit S Mozun R Kissoon N Watson RS . Susceptibility to childhood sepsis, contemporary management, and future directions. Lancet Child Adolesc Health. (2024) 8:682–94. doi: 10.1016/s2352-4642(24)00141-x 39142742

[7] Mwape RK Barday MA van der Zalm MM Verhagen LM . Overview of mucosal immunity and respiratory infections in children: a focus on Africa. Curr Opin Pediatr. (2025) 37:137–44. doi: 10.1097/mop.0000000000001438 39907513 PMC11888837

[8] Zhu W Wu Y Liu H Jiang C Huo L . Gut-lung axis: microbial crosstalk in pediatric respiratory tract infections. Front Immunol. (2021) 12:741233. doi: 10.3389/fimmu.2021.741233 34867963 PMC8637285

[9] Liu C Makrinioti H Saglani S Bowman M Lin LL Camargo CA Jr. . Microbial dysbiosis and childhood asthma development: Integrated role of the airway and gut microbiome, environmental exposures, and host metabolic and immune response. Front Immunol. (2022) 13:1028209. doi: 10.3389/fimmu.2022.1028209 36248891 PMC9561420

[10] Chiang CK Lai CL Chiu MH Huang CJ . The gut-lung axis in allergic asthma: A narrative review of microbial dysbiosis, immune regulation, and nutritional modulation. Nutrients. (2026) 18(9):1336. doi: 10.3390/nu18091336 42123938 PMC13164913

[11] Zhang C Zhang T Lu W Duan X Luo X Liu S . Altered airway microbiota composition in patients with pulmonary hypertension. Hypertension. (2020) 76:1589–99. doi: 10.1161/hypertensionaha.120.15025 32921193

[12] Perez-Garcia J Espuela-Ortiz A Hernández-Pérez JM González-Pérez R Poza-Guedes P Martin-Gonzalez E . Human genetics influences microbiome composition involved in asthma exacerbations despite inhaled corticosteroid treatment. J Allergy Clin Immunol. (2023) 152:799–806.e6. doi: 10.1016/j.jaci.2023.05.021 37301411 PMC10522330

[13] Claassen-Weitz S Gardner-Lubbe S Xia Y Mwaikono KS Mounaud SH Nierman WC . Succession and determinants of the early life nasopharyngeal microbiota in a South African birth cohort. Microbiome. (2023) 11:127. doi: 10.1186/s40168-023-01563-5 37271810 PMC10240772

[14] Azevedo AC Hilário S Gonçalves MFM . Microbiome in nasal mucosa of children and adolescents with allergic rhinitis: A systematic review. Children (Basel). (2023) 10(2):226. doi: 10.3390/children10020226 36832355 PMC9954962

[15] Sunde RB Thorsen J Kim M Schoos AM Stokholm J Bønnelykke K . Bacterial colonisation of the airway in neonates and risk of asthma and allergy until age 18 years. Eur Respir J. (2024) 63(1):2300471. doi: 10.1183/13993003.00471-2023 38097209

[16] Papadopoulos NG Apostolidou E Miligkos M Xepapadaki P . Bacteria and viruses and their role in the preschool wheeze to asthma transition. Pediatr Allergy Immunol. (2024) 35:e14098. doi: 10.1111/pai.14098 38445451

[17] Bodas-Pinedo A Lafuente EM Pelaez-Prestel HF Ras-Carmona A Subiza JL Reche PA . Combining different bacteria in vaccine formulations enhances the chance for antiviral cross-reactive immunity: a detailed in silico analysis for influenza A virus. Front Immunol. (2023) 14:1235053. doi: 10.3389/fimmu.2023.1235053 37675108 PMC10477994

[18] Bu ML Li MH Feng M Wang JR Sun L . Poly(I:C) exacerbates airway goblet cell hyperplasia and lung inflammation in HDM-exposed balb/C mice by YAP/FOXM1 pathway. Int Arch Allergy Immunol. (2023) 184:707–19. doi: 10.1159/000529109 36822170

[19] Carriera L Ielo S Barone R Lipsi R Coppola A Smargiassi A . High-flow nasal cannula in severe asthma exacerbations: current evidence and clinical perspectives. Eur Respir Rev. (2025) 34(178):250109. doi: 10.1183/16000617.0109-2025 41407390 PMC12709364

[20] McCauley KE DeMuri G Lynch K Fadrosh DW Santee C Nagalingam NN . Moraxella-dominated pediatric nasopharyngeal microbiota associate with upper respiratory infection and sinusitis. PloS One. (2021) 16:e0261179. doi: 10.1371/journal.pone.0261179 34962959 PMC8714118

[21] Claassen-Weitz S Xia Y Hannan L Gardner-Lubbe S Mwaikono KS Harris Mounaud S . Nasopharyngeal microbiota in South African infants with lower respiratory tract infection: A nested case-control study of the Drakenstein child health study. Clin Infect Dis. (2026) 81:e668–79. doi: 10.1093/cid/ciaf184 40241660 PMC13375580

[22] van Beveren GJ de Steenhuijsen Piters WAA Boeschoten SA Louman S Chu ML Arp K . Nasopharyngeal microbiota in children is associated with severe asthma exacerbations. J Allergy Clin Immunol. (2024) 153:1574–1585.e14. doi: 10.1016/j.jaci.2024.02.020 38467291

[23] Zimmermann P . Exploring the microbial landscape of the nasopharynx in children: a systematic review of studies using next generation sequencing. Front Microbiomes. (2023) 2:1231271. doi: 10.3389/frmbi.2023.1231271 41853378 PMC12993585

[24] Dicker AJ Huang JTJ Lonergan M Keir HR Fong CJ Tan B . The sputum microbiome, airway inflammation, and mortality in chronic obstructive pulmonary disease. J Allergy Clin Immunol. (2021) 147:158–67. doi: 10.1016/j.jaci.2020.02.040 32353489

[25] Lyly A Laulajainen-Hongisto A Gevaert P Kauppi P Toppila-Salmi S . Monoclonal antibodies and airway diseases. Int J Mol Sci. (2020) 21(24):9477. doi: 10.3390/ijms21249477 33322143 PMC7763928

[26] Wang C Xiao Z Cao Z Sheng F Xiang P Mu T . Fungal and bacterial pathogenic co-infections mainly lead to the assembly of microbial community in tobacco stems. Open Life Sci. (2025) 20:20251103. doi: 10.1515/biol-2025-1103 40989583 PMC12451430

[27] Xu L He R Ye X Wang Y Hui S Li H . Leveraging transcriptome-wide association studies identifies the relationship between upper respiratory flora and cell type-specific gene expression in severe respiratory disease. PloS One. (2025) 20:e0322864. doi: 10.1371/journal.pone.0322864 40343915 PMC12063895

[28] Goto T . Microbiota and lung cancer. Semin Cancer Biol. (2022) 86:1–10. doi: 10.1016/j.semcancer.2022.07.006 35882258

[29] Campbell S Gerasimidis K Milling S Dicker AJ Hansen R Langley RJ . The lower airway microbiome in paediatric health and chronic disease. Paediatr Respir Rev. (2024) 52:31–43. doi: 10.1016/j.prrv.2024.02.001 38538377

[30] Chen R Wang L Koch T Curtis V Yin-DeClue H Handley SA . Sex effects in the association between airway microbiome and asthma. Ann Allergy Asthma Immunol. (2020) 125:652–7.e3. doi: 10.1016/j.anai.2020.09.007 32931909 PMC8043253

[31] Oğuzülgen K Öztürk AB Bacceoğlu A Aydın Ö Köycü Buhari G Damadoğlu E . Inhaler steroid use changes oral and airway bacterial and fungal microbiome profile in asthma patients. Int Arch Allergy Immunol. (2024) 185:10–9. doi: 10.1159/000531866 37844548

[32] Kim YH Park MR Kim SY Kim MJ Kim KW Sohn MH . Respiratory microbiome profiles are associated with distinct inflammatory phenotype and lung function in children with asthma. J Investig Allergol Clin Immunol. (2024) 34:246–56. doi: 10.18176/jiaci.0918 37260034

[33] Kang SY Kim H Jung S Lee SM Lee SP . The lung microbiota in Korean patients with non-tuberculous mycobacterial pulmonary disease. BMC Microbiol. (2021) 21:84. doi: 10.1186/s12866-021-02141-1 33736609 PMC7977250

[34] Huang D Xie L Luo T Lin L Ren Q Zeng Z . Effects of azithromycin on alleviating airway inflammation in asthmatic mice by regulating airway microbiota and metabolites. Microbiol Spectr. (2025) 13:e0221724. doi: 10.1128/spectrum.02217-24 39932326 PMC11878009

[35] Boulund U Thorsen J Trivedi U Tranæs K Jiang J Shah SA . The role of the early-life gut microbiome in childhood asthma. Gut Microbes. (2025) 17:2457489. doi: 10.1080/19490976.2025.2457489 39882630 PMC11784655

[36] Kahhaleh FG Barrientos G Conrad ML . The gut-lung axis and asthma susceptibility in early life. Acta Physiol (Oxf). (2024) 240:e14092. doi: 10.1111/apha.14092 38251788

[37] Xue S Abdullahi R Wu N Zheng J Su M Xu M . Gut microecological regulation on bronchiolitis and asthma in children: A review. Clin Respir J. (2023) 17:975–85. doi: 10.1111/crj.13622 37105551 PMC10542989

[38] Mogoş GFR Manciulea Profir M Enache RM Pavelescu LA Popescu Roşu OA Cretoiu SM . Intestinal microbiota in early life: latest findings regarding the role of probiotics as a treatment approach for dysbiosis. Nutrients. (2025) 17(13):2071. doi: 10.3390/nu17132071 40647176 PMC12250897

[39] Zhao Y Wang H Lu Y Lou D . Evolving landscapes in childhood asthma-gut microbiota research: A bibliometric analysis from 2000 to 2024. Med (Baltimore). (2026) 105:e46594. doi: 10.1097/md.0000000000046594 41496069 PMC12778129

[40] Abdel-Aziz MI Hashimoto S Neerincx AH Haarman EG Cecil A Lintelmann J . Metabotypes are linked to uncontrolled childhood asthma, gut microbiota, and systemic inflammation. J Allergy Clin Immunol. (2025) 156:339–51. doi: 10.1016/j.jaci.2025.04.017 40280190

[41] Mann ER Lam YK Uhlig HH . Short-chain fatty acids: linking diet, the microbiome and immunity. Nat Rev Immunol. (2024) 24:577–95. doi: 10.1038/s41577-024-01014-8 38565643

[42] Wan J Song J Lv Q Zhang H Xiang Q Dai H . Alterations in the gut microbiome of young children with airway allergic disease revealed by next-generation sequencing. J Asthma Allergy. (2023) 16:961–72. doi: 10.2147/jaa.S422537 37700874 PMC10494927

[43] Peterson CT Perez Santiago J Iablokov SN Chopra D Rodionov DA Peterson SN . Short-chain fatty acids modulate healthy gut microbiota composition and functional potential. Curr Microbiol. (2022) 79:128. doi: 10.1007/s00284-022-02825-5 35287182 PMC8921067

[44] Steininger HM Iglesias-Aguirre CE Panzer AR Durack J McKean M Cabana MD . Carbohydrate metabolism differs in infants by asthma-risk status and is associated with the functional potential of bacteroides cellulosilyticus. bioRxiv. (2026) 2026.04.28.721144. doi: 10.64898/2026.04.28.721144 42146533 PMC13174680

[45] Lee-Sarwar KA Lasky-Su J Kelly RS Litonjua AA Weiss ST . Gut microbial-derived metabolomics of asthma. Metabolites. (2020) 10(3):97. doi: 10.3390/metabo10030097 32155960 PMC7142494

[46] Zimmermann-Rösner A Prehn-Kristensen A . The microbiome in child and adolescent psychiatry. Z Kinder Jugendpsychiatr Psychother. (2024) 52:213–26. doi: 10.1024/1422-4917/a000965 38240707

[47] Zhang J Zheng X Luo W Sun B . Cross-domain microbiomes: the interaction of gut, lung and environmental microbiota in asthma pathogenesis. Front Nutr. (2024) 11:1346923. doi: 10.3389/fnut.2024.1346923 38978703 PMC11229079

[48] Zhang M Qin Z Huang C Liang B Zhang X Sun W . The gut microbiota modulates airway inflammation in allergic asthma through the gut-lung axis related immune modulation: A review. Biomol BioMed. (2025) 25:727–38. doi: 10.17305/bb.2024.11280 39465678 PMC11959394

[49] Dasgupta S . Unraveling the microbiome-asthma axis: metagenomic insights from airway and gut microbial communities. Omics. (2025) 29:374–83. doi: 10.1177/15578100251358958 40638549

[50] Fischer-RasmussenK GranellR EliasenAU KreinerE PedersenCT LuoY . Genetic characterization of preschool wheeze phenotypes. J Allergy Clin Immunol. (2025) 156:1537–46. doi: 10.1016/j.jaci.2025.07.015 40769318

[51] Pichler WJ Brüggen MC . Viral infections and drug hypersensitivity. Allergy. (2023) 78:60–70. doi: 10.1111/all.15558 36264263

[52] Sim YS Lee JH Lee EG Choi JY Lee CH An TJ . COPD exacerbation-related pathogens and previous COPD treatment. J Clin Med. (2022) 12(1):111. doi: 10.3390/jcm12010111 36614912 PMC9821136

[53] Fouka E Domvri K Gkakou F Alevizaki M Steiropoulos P Papakosta D . Recent insights in the role of biomarkers in severe asthma management. Front Med (Lausanne). (2022) 9:992565. doi: 10.3389/fmed.2022.992565 36226150 PMC9548530

[54] Zheng P Zhang K Lv X Liu C Wang Q Bai X . Gut microbiome and metabolomics profiles of allergic and non-allergic childhood asthma. J Asthma Allergy. (2022) 15:419–35. doi: 10.2147/jaa.S354870 35418758 PMC8995180

[55] Yang W Ren D Zhao Y Liu L Yang X . Fuzhuan brick tea polysaccharide improved ulcerative colitis in association with gut microbiota-derived tryptophan metabolism. J Agric Food Chem. (2021) 69:8448–59. doi: 10.1021/acs.jafc.1c02774 34313113

[56] Chen XL Cai K Zhang W Su SL Zhao LH Qiu LP . Bear bile powder ameliorates type 2 diabetes via modulation of metabolic profiles, gut microbiota, and metabolites. Front Pharmacol. (2022) 13:1090955. doi: 10.3389/fphar.2022.1090955 36686652 PMC9846258

[57] Yu B Pei C Peng W Zheng Y Fu Y Wang X . Microbiota-derived butyrate alleviates asthma via inhibiting Tfh13-mediated IgE production. Signal Transduct Target Ther. (2025) 10:181. doi: 10.1038/s41392-025-02263-2 40473603 PMC12141656

[58] Mo M Chen L Wang Y Lin X Li H Chen B . The gut-lung axis in childhood asthma: from early-life programming to microbiome-informed precision medicine-a narrative review. Front Immunol. (2026) 17:1814901. doi: 10.3389/fimmu.2026.1814901 42099620 PMC13143597

[59] Ma Y Zheng H Tian H Cui J Liu L . Research advance in correlation between childhood asthma and gut microbiota. Front Cell Infect Microbiol. (2025) 15:1649180. doi: 10.3389/fcimb.2025.1649180 41122685 PMC12536028

[60] Gisevius B Duscha A Poschmann G Stühler K Motte J Fisse AL . Propionic acid promotes neurite recovery in damaged multiple sclerosis neurons. Brain Commun. (2024) 6:fcae182. doi: 10.1093/braincomms/fcae182 38894951 PMC11184351

[61] Abdalkareem Jasim S Jade Catalan Opulencia M Alexis Ramírez-Coronel A Kamal Abdelbasset W Hasan Abed M Markov A . The emerging role of microbiota-derived short-chain fatty acids in immunometabolism. Int Immunopharmacol. (2022) 110:108983. doi: 10.1016/j.intimp.2022.108983 35750016

[62] Lan F Zhang N Bachert C Zhang L . Stability of regulatory T cells in T helper 2-biased allergic airway diseases. Allergy. (2020) 75:1918–26. doi: 10.1111/all.14257 32124987

[63] Sun Y He M Li J Wu C Pan S . Short-chain fatty acids: A key modulator of sepsis-associated acute respiratory distress syndrome. J Inflammation Res. (2026) 19:608128. doi: 10.2147/jir.S608128 42345000 PMC13288758

[64] Yamamura R Nakamura K Kitada N Aizawa T Shimizu Y Nakamura K . Associations of gut microbiota, dietary intake, and serum short-chain fatty acids with fecal short-chain fatty acids. Biosci Microbiota Food Health. (2020) 39:11–7. doi: 10.12938/bmfh.19-010 32010539 PMC6971417

[65] Morillas-Armenta J Macías-Camero A Rzetecka N Zubeldia-Varela E Barker-Tejeda TC Escribese MM . Microbial metabolites in allergic diseases: Beyond short-chain fatty acids. Curr Allergy Asthma Rep. (2025) 25:53. doi: 10.1007/s11882-025-01231-8 41214356

[66] Lejeune S Kaushik A Parsons ES Chinthrajah S Snyder M Desai M . Untargeted metabolomic profiling in children identifies novel pathways in asthma and atopy. J Allergy Clin Immunol. (2024) 153:418–34. doi: 10.1016/j.jaci.2023.09.040 38344970

[67] Mar JS Ota N Pokorzynski ND Peng Y Jaochico A Sangaraju D . IL-22 alters gut microbiota composition and function to increase aryl hydrocarbon receptor activity in mice and humans. Microbiome. (2023) 11:47. doi: 10.1186/s40168-023-01486-1 36894983 PMC9997005

[68] Torrelli-Diljohn A Kulkarni B Vitturi DA . Cell signaling by tryptophan catabolism. Biochemistry. (2026) 65:1366–94. doi: 10.1021/acs.biochem.6c00179 42029070 PMC13151070

[69] Łukawska A Mulak A . Impact of primary and secondary bile acids on Clostridioides difficile infection. Pol J Microbiol. (2022) 71:11–8. doi: 10.33073/pjm-2022-007 35635171 PMC9152914

[70] Yu J Li S Guo J Xu Z Zheng J Sun X . Farnesoid X receptor antagonizes Wnt/β-catenin signaling in colorectal tumorigenesis. Cell Death Dis. (2020) 11:640. doi: 10.1038/s41419-020-02819-w 32807788 PMC7431544

[71] Su X Gao Y Yang R . Gut microbiota derived bile acid metabolites maintain the homeostasis of gut and systemic immunity. Front Immunol. (2023) 14:1127743. doi: 10.3389/fimmu.2023.1127743 37256134 PMC10225537

[72] Ghaffari MH Sanz-Fernandez MV Sadri H Sauerwein H Schuchardt S Martín-Tereso J . Longitudinal characterization of the metabolome of dairy cows transitioning from one lactation to the next: Investigations in the liver. J Dairy Sci. (2024) 107:4000–16. doi: 10.3168/jds.2023-24432 38246557

[73] Rzetecka N Matysiak J Matysiak J Sobkowiak P Wojsyk-Banaszak I Bręborowicz A . Metabolomics in childhood asthma - a promising tool to meet various clinical needs. Curr Allergy Asthma Rep. (2025) 25:24. doi: 10.1007/s11882-025-01198-6 40341431 PMC12062110

[74] Ma S Hu W Bi Y Han Y Wang W Xin D . Exploring the causal role of plasma metabolites in pediatric asthma: a Mendelian randomization study. J Asthma. (2025) 62:2070–83. doi: 10.1080/02770903.2025.2552748 40905282

[75] Wang YH Limaye A Liu JR Wu TN . Potential probiotics for regulation of the gut-lung axis to prevent or alleviate influenza in vulnerable populations. J Tradit Complement Med. (2023) 13:161–9. doi: 10.1016/j.jtcme.2022.08.004 36970463 PMC10037066

[76] Zhu Y Ding RD . Role of the gut-lung axis in bronchopulmonary dysplasia: Physiological basis, pathogenesis and immunological modulation (review). Mol Med Rep. (2025) 32(6):313. doi: 10.3892/mmr.2025.13678 40937590 PMC12447068

[77] Azeez A Baugh JA . The role of dietary fibre in lung inflammation: microbiota, metabolites, and immune crosstalk. Inflammation Res. (2025) 74:135. doi: 10.1007/s00011-025-02098-1 41042335

[78] Luo W Li R Pan C Luo C . Gut microbiota-derived metabolites in immunomodulation and gastrointestinal cancer immunotherapy. Front Immunol. (2025) 16:1710880. doi: 10.3389/fimmu.2025.1710880 41357193 PMC12678330

[79] Porbahaie M Hummel A Saouadogo H Coelho RML Savelkoul HFJ Teodorowicz M . Short-chain fatty acids inhibit the activation of T lymphocytes and myeloid cells and induce innate immune tolerance. Benef Microbes. (2023) 14:401–19. doi: 10.1163/18762891-20220113 38661366

[80] Duan H Wang L Huangfu M Li H . The impact of microbiota-derived short-chain fatty acids on macrophage activities in disease: Mechanisms and therapeutic potentials. BioMed Pharmacother. (2023) 165:115276. doi: 10.1016/j.biopha.2023.115276 37542852

[81] Yuan S Bremmer A Yang X Hu Q . Microbiota metabolism and immune regulation: From mechanisms to immunotherapeutic applications in cancer. Semin Cancer Biol. (2025) 117:168–83. doi: 10.1016/j.semcancer.2025.11.023 41270950

[82] Song XL Liang J Lin SZ Xie YW Ke CH Ao D . Gut-lung axis and asthma: A historical review on mechanism and future perspective. Clin Transl Allergy. (2024) 14:e12356. doi: 10.1002/clt2.12356 38687096 PMC11060082

[83] Pattaroni C Marsland BJ Harris NL . Early-life host-microbial interactions and asthma development: A lifelong impact? Immunol Rev. (2025) 330:e70019. doi: 10.1111/imr.70019 40099971 PMC11917194

[84] Zhang X Zhang Z Peng Q Huang X Liu M Bai D . The immunogenetic landscape of allergic rhinitis: from cellular effectors to gene regulation and targeted therapies. Int J Biol Sci. (2026) 22:1322–45. doi: 10.7150/ijbs.126788 41608632 PMC12837821

[85] Brüggemann TR Krishnamoorthy N Hagner M Matschiner G Jaquin T Tavares LP . A new Anticalin protein for IL-23 inhibits non-type 2 allergen-driven mouse lung inflammation and airway hyperresponsiveness. Am J Physiol Lung Cell Mol Physiol. (2024) 327:L624–l633. doi: 10.1152/ajplung.00295.2023 39104317 PMC11563638

[86] Dudhat K . Physical activity increases immunity to COVID-19 infection. Crit Rev Immunol. (2023) 43:1–10. doi: 10.1615/CritRevImmunol.2023049460 37831519

[87] Parrón-Ballesteros J Gordo RG López-Rodríguez JC Olmo N Villalba M Batanero E . Beyond allergic progression: From molecules to microbes as barrier modulators in the gut-lung axis functionality. Front Allergy. (2023) 4:1093800. doi: 10.3389/falgy.2023.1093800 36793545 PMC9923236

[88] Du B Fu Y Han Y Sun Q Xu J Yang Y . The lung-gut crosstalk in respiratory and inflammatory bowel disease. Front Cell Infect Microbiol. (2023) 13:1218565. doi: 10.3389/fcimb.2023.1218565 37680747 PMC10482113

[89] Mazumder MHH Hussain S . Air-pollution-mediated microbial dysbiosis in health and disease: Lung-gut axis and beyond. J Xenobiot. (2024) 14:1595–612. doi: 10.3390/jox14040086 39449427 PMC11503347

[90] Gasaly N de Vos P Hermoso MA . Impact of bacterial metabolites on gut barrier function and host immunity: A focus on bacterial metabolism and its relevance for intestinal inflammation. Front Immunol. (2021) 12:658354. doi: 10.3389/fimmu.2021.658354 34122415 PMC8187770

[91] Zhu J He M Li S Lei Y Xiang X Guo Z . Shaping oral and intestinal microbiota and the immune system during the first 1,000 days of life. Front Pediatr. (2025) 13:1471743. doi: 10.3389/fped.2025.1471743 39906673 PMC11790674

[92] Okoshi K Sakurai K Yamamoto M Mori C . Maternal antibiotic exposure and childhood allergies: The Japan Environment and Children's Study. J Allergy Clin Immunol Glob. (2023) 2:100137. doi: 10.1016/j.jacig.2023.100137 37781654 PMC10509907

[93] Ren H Wu Q Sun Z Fang M Liu J Luo J . Research progress and treatment of radiation enteritis and gut microbiota. Radiat Oncol J. (2023) 41:61–8. doi: 10.3857/roj.2023.00346 37403348 PMC10326510

[94] Kim NH Choi BY Kim ES Kim SJ Hong JY Heo SH . Systemic antibiotics cause deterioration of emphysema associated with exaggerated inflammation and autophagy. Exp Mol Med. (2023) 55:2260–8. doi: 10.1038/s12276-023-01099-6 37779147 PMC10618248

[95] Wurm J Curtis N Zimmermann P . The effect of antibiotics on the intestinal microbiota in children - a systematic review. Front Allergy. (2024) 5:1458688. doi: 10.3389/falgy.2024.1458688 39435363 PMC11491438

[96] Lee E Park YM Lee SY Lee SH Park MJ Ahn K . Associations of prenatal antibiotic exposure and delivery mode on childhood asthma inception. Ann Allergy Asthma Immunol. (2023) 131:52–58.e1. doi: 10.1016/j.anai.2023.03.020 36990205

[97] Gestels T Vandenplas Y . Prenatal and perinatal antibiotic exposure and long-term outcome. Pediatr Gastroenterol Hepatol Nutr. (2023) 26:135–45. doi: 10.5223/pghn.2023.26.3.135 37214166 PMC10192590

[98] Toivonen L Schuez-Havupalo L Karppinen S Waris M Hoffman KL Camargo CA . Antibiotic treatments during infancy, changes in nasal microbiota, and asthma development: Population-based cohort study. Clin Infect Dis. (2021) 72:1546–54. doi: 10.1093/cid/ciaa262 32170305 PMC8096219

[99] Kyvsgaard JN Brustad N Hesselberg LM Vahman N Thorsen J Schoos AM . Key risk factors of asthma-like symptoms are mediated through infection burden in early childhood. J Allergy Clin Immunol. (2024) 153:684–94. doi: 10.1016/j.jaci.2023.11.019 37995855

[100] Kesäläinen A Rantanen R Honkila M Helminen M Rahkonen O Kallio M . Effects of antibiotics, hospitalisation and surgical complications on self-reported immunological vulnerability following paediatric open-heart surgery and thymectomy: a single-centre retrospective cohort study. BMJ Paediatr Open. (2024) 8(1):e002651. doi: 10.1136/bmjpo-2024-002651 38830724 PMC11149146

[101] Giordani B Abruzzo A Parolin C Foschi C Laghi L Marangoni A . Prebiotic activity of vaginal lactobacilli on bifidobacteria: from concept to formulation. Microbiol Spectr. (2023) 11:e0200922. doi: 10.1128/spectrum.02009-22 36602371 PMC9927276

[102] Rapin A Rehbinder EM Macowan M Pattaroni C Lødrup Carlsen KC Harris NL . The skin microbiome in the first year of life and its association with atopic dermatitis. Allergy. (2023) 78:1949–63. doi: 10.1111/all.15671 36779606

[103] Subramanian P Romero-Soto HN Stern DB Maxwell GL Levy S Hourigan SK . Delivery mode impacts gut bacteriophage colonization during infancy. Gut Microbes Rep. (2025) 2(1):2464631. doi: 10.1080/29933935.2025.2464631 40823537 PMC12352455

[104] Moore LE Serrano-Lomelin J Rosychuk RJ Kozyrskyj AL Chari R Crawford S . Perinatal and early life factors and asthma control among preschoolers: a population-based retrospective cohort study. BMJ Open Respir Res. (2023) 10(1):e001928. doi: 10.1136/bmjresp-2023-001928 37748808 PMC10533801

[105] Davaanyam E Otgonbaatar U Nakajima A Sakanaka M Ojima MN Kozakai T . Human milk oligosaccharides and infant gut microbiome in Mongolian mother-infant dyads. J Dairy Sci. (2025) 108:11871–88. doi: 10.3168/jds.2025-26865 40912567

[106] Tian M Li Q Zheng T Yang S Chen F Guan W . Maternal microbe-specific modulation of the offspring microbiome and development during pregnancy and lactation. Gut Microbes. (2023) 15:2206505. doi: 10.1080/19490976.2023.2206505 37184203 PMC10187089

[107] Demay MB Pittas AG Bikle DD Diab DL Kiely ME Lazaretti-Castro M . Vitamin D for the prevention of disease: An Endocrine Society Clinical Practice Guideline. J Clin Endocrinol Metab. (2024) 109:1907–47. doi: 10.1210/clinem/dgae290 38828931

[108] Alswat AS . The influence of the gut microbiota on host health: A focus on the gut-lung axis and therapeutic approaches. Life (Basel). (2024) 14(10):1279. doi: 10.3390/life14101279 39459579 PMC11509314

[109] Venter C Smith PK Arshad H . Dietary strategies for the prevention of asthma in children. Curr Opin Allergy Clin Immunol. (2022) 22:123–31. doi: 10.1097/aci.0000000000000805 35197434

[110] Zakarya R Chan YL Wang B Thorpe A Xenaki D Ho KF . Developmental air pollution exposure augments airway hyperreactivity, alters transcriptome, and DNA methylation in female adult progeny. Commun Biol. (2025) 8:400. doi: 10.1038/s42003-025-07835-0 40057553 PMC11890619

[111] DeKruyff RH Zhang W Nadeau KC Leung DYM Wills-Karp M . Summary of the Keystone Symposium "Origins of allergic disease: Microbial, epithelial and immune interactions," March 24-27, Tahoe City, California. J Allergy Clin Immunol. (2020) 145:1072–81.e1. doi: 10.1016/j.jaci.2019.11.048 31926182

[112] Künstle N Gorlanova O Rüttimann C Mostacci N Röösli M de Hoogh K . The association of increased pre- and postnatal NO(2) and PM(2.5) exposure with the infant nasal microbiome composition and respiratory symptoms. Environ Res. (2025) 267:120694. doi: 10.1016/j.envres.2024.120694 39725140

[113] Le Souëf PN Adachi Y Anastasiou E Ansotegui IJ Badellino HA Banzon T . Global change, climate change, and asthma in children: Direct and indirect effects - A WAO Pediatric Asthma Committee Report. World Allergy Organ J. (2024) 17:100988. doi: 10.1016/j.waojou.2024.100988 39582513 PMC11584610

[114] Kim BE Hui-Beckman JW Nevid MZ Goleva E Leung DYM . Air pollutants contribute to epithelial barrier dysfunction and allergic diseases. Ann Allergy Asthma Immunol. (2024) 132:433–9. doi: 10.1016/j.anai.2023.11.014 38006973

[115] Sanidad KZ Rager SL Carrow HC Ananthanarayanan A Callaghan R Hart LR . Gut bacteria-derived serotonin promotes immune tolerance in early life. Sci Immunol. (2024) 9:eadj4775. doi: 10.1126/sciimmunol.adj4775 38489352 PMC11328322

[116] Chen Z Zhang L Ai T Fan Y Liu Y Wang L . Air pollution and childhood asthma hospitalizations in Chengdu, China: A time-series study. J Asthma Allergy. (2025) 18:229–43. doi: 10.2147/jaa.S498234 39990055 PMC11846614

[117] Zhang ZQ Li JY Guo Q Li YL Bao YW Song YQ . Association between air pollution and allergic upper respiratory diseases: a meta-analysis. Eur Respir Rev. (2025) 34(176):240266. doi: 10.1183/16000617.0266-2024 40562438 PMC12220745

[118] Cheng SL Hedges M Keski-Rahkonen P Chatziioannou AC Scalbert A Chung KF . Multiomic signatures of traffic-related air pollution in London reveal potential short-term perturbations in gut microbiome-related pathways. Environ Sci Technol. (2024) 58:8771–82. doi: 10.1021/acs.est.3c09148 38728551 PMC11112755

[119] Liu J Zhang S Xie H Lu J Zhang Z Xie Q . Integrated screening and risk prioritization of SVOCs in indoor air across commercial and residential environments. J Hazard Mater. (2026) 503:141209. doi: 10.1016/j.jhazmat.2026.141209 41592412

[120] Vercelli D . From Amish farm dust to bacterial lysates: The long and winding road to protection from allergic disease. Semin Immunol. (2023) 68:101779. doi: 10.1016/j.smim.2023.101779 37210851 PMC10330614

[121] Xing Y Tsang MS Yang Z Wang MH Pivniouk V Leung AS . Immune modulation by rural exposures and allergy protection. Pediatr Allergy Immunol. (2024) 35:e14086. doi: 10.1111/pai.14086 38351891

[122] Tessin J Jung A Silberborth A Rohn K Schulz J Visscher C . Detection of Enterococcus cecorum to identify persistently contaminated locations using faecal and environmental samples in broiler houses of clinically healthy flocks. Avian Pathol. (2024) 53:312–20. doi: 10.1080/03079457.2024.2334682 38525653

[123] Mahmud B Vargas RC Sukhum KV Patel S Liao J Hall LR . Longitudinal dynamics of farmer and livestock nasal and faecal microbiomes and resistomes. Nat Microbiol. (2024) 9:1007–20. doi: 10.1038/s41564-024-01639-4 38570675 PMC11966613

[124] Kelly MS Bunyavanich S Phipatanakul W Lai PS . The environmental microbiome, allergic disease, and asthma. J Allergy Clin Immunol Pract. (2022) 10:2206–2217.e1. doi: 10.1016/j.jaip.2022.06.006 35750322 PMC9704440

[125] Fan D Hu J Lin N . Effects of probiotics, prebiotics, synbiotics and postbiotics on pediatric asthma: a systematic review. Front Nutr. (2025) 12:1586129. doi: 10.3389/fnut.2025.1586129 40352259 PMC12061971

[126] Beswick G Major N Hendrickson C Kumar V Waller M Durrani Z . A scoping review on the role of the microbiome as a factor in the bidirectional association between obesity and depression. Curr Diabetes Rep. (2025) 25:56. doi: 10.1007/s11892-025-01607-0 41160345

[127] Chen X Yong SB Yii CY Feng B Hsieh KS Li Q . Intestinal microbiota and probiotic intervention in children with bronchial asthma. Heliyon. (2024) 10:e34916. doi: 10.1016/j.heliyon.2024.e34916 39144926 PMC11320201

[128] Kleniewska P Pawliczak R . Can probiotics be used in the prevention and treatment of bronchial asthma? Pharmacol Rep. (2024) 76:740–53. doi: 10.1007/s43440-024-00618-0 38951480 PMC11294272

[129] Hmar EBL Paul S Sharma HK . An insight into the combination of probiotics and their implications for human health. Endocr Metab Immune Disord Drug Targets. (2024) 24:1–12. doi: 10.2174/1871530323666230502141717 37138440

[130] Sproten R Nohr D Guseva D . Nutritional strategies modulating the gut microbiome as a preventative and therapeutic approach in normal and pathological age-related cognitive decline: a systematic review of preclinical and clinical findings. Nutr Neurosci. (2024) 27:1042–57. doi: 10.1080/1028415x.2023.2296727 38165747

[131] Dorosky RJ Schreier JE Lola SL Sava RL Coryell MP Akue A . Nanobodies as potential tools for microbiological testing of live biotherapeutic products. AMB Express. (2024) 14:9. doi: 10.1186/s13568-023-01659-z 38245586 PMC10799837

[132] Liu Z Bai P Wang L Zhu L Zhu Z Jiang L . Clostridium tyrobutyricum in combination with chito-oligosaccharides modulate inflammation and gut microbiota for inflammatory bowel disease treatment. J Agric Food Chem. (2024) 72:18497–506. doi: 10.1021/acs.jafc.4c03486 39099138

[133] Wang L Xu L . The impact of prebiotics, probiotics and synbiotics on the prevention and treatment of atopic dermatitis in children: an umbrella meta-analysis. Front Pediatr. (2025) 13:1498965. doi: 10.3389/fped.2025.1498965 40191649 PMC11968740

[134] Trisal A Singh I Garg G Jorwal K Singh AK . Gut-brain axis and brain health: modulating neuroinflammation, cognitive decline, and neurodegeneration. 3 Biotech. (2025) 15:25. doi: 10.1007/s13205-024-04187-0 39735610 PMC11680542

[135] Deligeorgopoulou M Tsabouri S Siomou E Vlahos AP Serbis A . Dietary habits and their impact on pediatric obesity and asthma: A narrative review with emphasis on the Mediterranean diet. Children (Basel). (2025) 12(10):1354. doi: 10.3390/children12101354 41153538 PMC12563717

[136] Ren H Dong S Li L Zhao W . Effects of soluble and insoluble fibre on glycolipid metabolism and gut microbiota in high-fat-diet-induced obese mice. Nutrients. (2024) 16(22):3822. doi: 10.3390/nu16223822 39599608 PMC11597833

[137] Martinez-Medina JN Flores-Lopez R López-Contreras BE Villamil-Ramirez H Guzman-Muñoz D Macias-Kauffer LR . Effect of gut microbial enterotypes on the association between habitual dietary fiber intake and insulin resistance markers in Mexican children and adults. Nutrients. (2021) 13(11):3892. doi: 10.3390/nu13113892 34836148 PMC8625328

[138] Kochumon S Madhoun AA Al-Rashed F Azim R Al-Ozairi E Al-Mulla F . Adipose tissue gene expression of CXCL10 and CXCL11 modulates inflammatory markers in obesity: implications for metabolic inflammation and insulin resistance. Ther Adv Endocrinol Metab. (2020) 11:2042018820930902. doi: 10.1177/2042018820930902 32655851 PMC7331767

[139] Kundi ZM Lee JC Pihlajamäki J Chan CB Leung KS So SSY . Dietary fiber from oat and rye brans ameliorate Western diet-induced body weight gain and hepatic inflammation by the modulation of short-chain fatty acids, bile acids, and tryptophan metabolism. Mol Nutr Food Res. (2021) 65:e1900580. doi: 10.1002/mnfr.201900580 32526796

[140] Katz DL Gardner CD . Perspective: Nutrition research & programming in multicultural populations: The fixed-quality variable-type (FQVT) dietary intervention. Adv Nutr. (2025) 16:100505. doi: 10.1016/j.advnut.2025.100505 40914208 PMC12592231

[141] Dai DLY Manus MB Hoskinson C Jiang J Sbihi H Miliku K . Breastfeeding may lessen socioeconomic disparities in child health through differences in the infant gut microbiome. Cell Rep Med. (2026) 7:102755. doi: 10.1016/j.xcrm.2026.102755 41980780 PMC13130633

[142] Donald K Serapio-Palacios A Bozorgmehr T Ma M Garcia MAI Petersen C . Human milk IgA promotes normal immune development by limiting Th17-inducing Erysipelatoclostridium ramosum in the infant gut. Proc Natl Acad Sci USA. (2025) 122:e2501030122. doi: 10.1073/pnas.2501030122 40623174 PMC12280908

[143] Wang W Tu M Huang L Zhang X Chen X Lin L . Association of breastfeeding practices during the first 12 months and subsequent infant respiratory tract infections: a prospective cohort study. Eur J Clin Nutr. (2025) 79:345–50. doi: 10.1038/s41430-024-01558-x 39706879

[144] Vassilopoulou E Agostoni C Feketea G Alberti I Gianni ML Milani GP . The role of breastfeeding in acute respiratory infections in infancy. Pediatr Infect Dis J. (2024) 43:1090–9. doi: 10.1097/inf.0000000000004454 38986006

[145] Zhao G Xie L Wu Y Wang B Teng W Sun Z . Effects of urbanization and lifestyle habits on the intestinal microbiota of adolescents in eastern China. Front Microbiol. (2023) 14:989303. doi: 10.3389/fmicb.2023.989303 37378282 PMC10291051

[146] Su H Xie H . Associations between lifestyle habits, environmental factors and respiratory diseases: a cross-sectional study from southwest China. Front Public Health. (2025) 13:1513926. doi: 10.3389/fpubh.2025.1513926 40109413 PMC11919831

[147] Yu Y Wang W Zhang F . The next generation fecal microbiota transplantation: To transplant bacteria or virome. Adv Sci (Weinh). (2023) 10:e2301097. doi: 10.1002/advs.202301097 37914662 PMC10724401

[148] Liu C Dan L Wang X Chen L Yuan X . Gut microbiota impact on lung diseases: a mini review of clinical evidence. Infect Immun. (2026) 94:e0043025. doi: 10.1128/iai.00430-25 41874370 PMC13081720

[149] Liu D Hu L Yang Y Wang Y Li Y Su J . Saccharomyces boulardii alleviates allergic asthma by restoring gut microbiota and metabolic homeostasis via up-regulation of METTL3 in an m6A-dependent manner. Immunol Lett. (2024) 267:106853. doi: 10.1016/j.imlet.2024.106853 38513836

[150] Dey P Ray Chaudhuri S . The opportunistic nature of gut commensal microbiota. Crit Rev Microbiol. (2023) 49:739–63. doi: 10.1080/1040841x.2022.2133987 36256871

[151] Wetthasinghe L Ng HF Chew KS Ranai NM Ngeow YF Lee WS . Paediatric Crohn's disease management: A mini review exploring conventional and innovative therapies with promising potential. J Gastroenterol Hepatol. (2026) 41:1204–12. doi: 10.1111/jgh.70307 41705405

[152] Lee TY Sadatsafavi M Yadav CP Price DB Beasley R Janson C . Individualised risk prediction model for exacerbations in patients with severe asthma: Protocol for a multicentre real-world risk modelling study. BMJ Open. (2023) 13:e070459. doi: 10.1136/bmjopen-2022-070459 36894199 PMC10008482

[153] Tiwari R Paswan A Tiwari G Reddy VJS Posa MK . Perspectives on fecal microbiota transplantation: Uses and modes of administration. Zhongguo Ying Yong Sheng Li Xue Za Zhi. (2025) 41:e20250014. doi: 10.62958/j.cjap.2025.014 40589142

[154] Xu H Wang X Feng W Liu Q Zhou S Liu Q . The gut microbiota and its interactions with cardiovascular disease. Microb Biotechnol. (2020) 13:637–56. doi: 10.1111/1751-7915.13524 31984651 PMC7111081

[155] Juonala M Wu F Sinaiko A Woo JG Urbina EM Jacobs D . Non-HDL cholesterol levels in childhood and carotid intima-media thickness in adulthood. Pediatrics. (2020) 145(4):e20192114. doi: 10.1542/peds.2019-2114 32209701 PMC7111486

[156] Gallitto G Englert R Kincses B Kotikalapudi R Li J Hoffschlag K . External validation of machine learning models-registered models and adaptive sample splitting. GigaScience. (2025) 14:giaf036. doi: 10.1093/gigascience/giaf036 40366867 PMC12077397

[157] Ikeda T Nishida A Yamano M Kimura I . Short-chain fatty acid receptors and gut microbiota as therapeutic targets in metabolic, immune, and neurological diseases. Pharmacol Ther. (2022) 239:108273. doi: 10.1016/j.pharmthera.2022.108273 36057320

[158] Zhu M Liu X Jing Q Yao L Yao X . Skin-lung axis in the atopic march: The immune link from skin to lungs. Chin Med J Pulm Crit Care Med. (2025) 3:273–88. doi: 10.1016/j.pccm.2025.11.002 41551010 PMC12805404

[159] Jacques C Floris I . How an immune-factor-based formulation of micro-immunotherapy could interfere with the physiological processes involved in the atopic march. Int J Mol Sci. (2023) 24(2):1483. doi: 10.3390/ijms24021483 36675006 PMC9864899

[160] Yuan J Yu Z Wang X Feng L Tang J Li Z . The research progress on the relationship between asthma and skin barrier damage. Br J Hosp Med (Lond). (2026) 87:50386. doi: 10.31083/bjhm50386 41609150

[161] Choi EH Kang H . Importance of stratum corneum acidification to restore skin barrier function in eczematous diseases. Ann Dermatol. (2024) 36:1–8. doi: 10.5021/ad.23.078 38325428 PMC10861303

[162] Taki MH Lee KE Gangnon R Gern JE Lemanske RF Jr. Jackson D . Atopic dermatitis phenotype affects expression of atopic diseases despite similar mononuclear cell cytokine response. J Allergy Clin Immunol. (2024) 153:1604–10.e2. doi: 10.1016/j.jaci.2024.02.015 38438085 PMC11956522

[163] Demessant-Flavigny AL Connétable S Kerob D Moreau M Aguilar L Wollenberg A . Skin microbiome dysbiosis and the role of Staphylococcus aureus in atopic dermatitis in adults and children: A narrative review. J Eur Acad Dermatol Venereol. (2023) 37:3–17. doi: 10.1111/jdv.19125 37232427

[164] Chehadeh C Nakatsuji T Gallo RL . Staphylococcus aureus in atopic dermatitis: How a common bacterium exploits and drives disease. J Allergy Clin Immunol. (2026) 157:551–7. doi: 10.1016/j.jaci.2025.12.1009 41539578 PMC13476856

[165] Taico Oliva C Musa I Kopulos D Ardalani F Maskey A Wilson A . The gut microbiome and cross-reactivity of food allergens: Current understanding, insights, and future directions. Front Allergy. (2024) 5:1503380. doi: 10.3389/falgy.2024.1503380 39872379 PMC11769990

[166] Pachauri A Sharma S . Unravelling the gut-skin axis: The role of gut microbiota in pathogenesis and management of psoriasis. Inflammopharmacology. (2025) 33:3671–8. doi: 10.1007/s10787-025-01813-y 40504322

[167] Yang L Lin Z Gao T Wang P Wang G . The role of skin-gut-lung microbiome in allergic diseases. J Allergy Clin Immunol Pract. (2025) 13:1935–42.e4. doi: 10.1016/j.jaip.2025.04.041 40320144

[168] Santos EA Silva JL Leocádio PCL Andrade MER Queiroz-Junior CM Oliveira N . Cutaneous application of capsaicin cream reduces clinical signs of experimental colitis and repairs intestinal barrier integrity by modulating the gut microbiota and tight junction proteins. ACS Pharmacol Transl Sci. (2024) 7:2143–53. doi: 10.1021/acsptsci.4c00207 39022369 PMC11249629

[169] Verma A Bhagchandani T Rai A Nikita Sardarni UK Bhavesh N . Short-chain fatty acid (SCFA) as a connecting link between microbiota and gut-lung axis-a potential therapeutic intervention to improve lung health. ACS Omega. (2024) 9:14648–71. doi: 10.1021/acsomega.3c05846 38585101 PMC10993281

